# Photocarrier drift distance in organic solar cells and photodetectors

**DOI:** 10.1038/srep09949

**Published:** 2015-04-28

**Authors:** Martin Stolterfoht, Ardalan Armin, Bronson Philippa, Ronald D. White, Paul L. Burn, Paul Meredith, Gytis Juška, Almantas Pivrikas

**Affiliations:** 1 Centre For Organic Photonics & Electronics (COPE), School of Chemistry and Molecular Biosciences and School of Mathematics and Physics, The University of Queensland, Brisbane 4072, Australia; 2 College of Science, Technology and Engineering, James Cook University, Townsville 4811, Australia; 3 Department of Solid State Electronics Vilnius University 10222 Vilnius, Lithuania; 4 School of Engineering and Information Technology, Murdoch University, Perth 6150, Australia

## Abstract

Light harvesting systems based upon disordered materials are not only widespread in nature, but are also increasingly prevalent in solar cells and photodetectors. Examples include organic semiconductors, which typically possess low charge carrier mobilities and Langevin-type recombination dynamics – both of which negatively impact the device performance. It is accepted wisdom that the “drift distance” (i.e., the distance a photocarrier drifts before recombination) is defined by the mobility-lifetime product in solar cells. We demonstrate that this traditional figure of merit is inadequate for describing the charge transport physics of organic light harvesting systems. It is experimentally shown that the onset of the photocarrier recombination is determined by the electrode charge and we propose the mobility-recombination coefficient product as an alternative figure of merit. The implications of these findings are relevant to a wide range of light harvesting systems and will necessitate a rethink of the critical parameters of charge transport.

Light harvesting devices fabricated using non-single-crystal films such as polymers, organic molecules, dye-sensitized structures, nanoparticles as well as perovskites offer the potential for low cost and large area fabrication. All these systems lack long-range electronic order and have a common feature, i.e., their electrical conduction is inferior to highly-crystalline inorganic semiconductors such as silicon. The relatively poor electrical conduction arises because of their orders of magnitude lower electron and hole mobilities, and the low density of intrinsic charge carriers. The low photocarrier mobility causes charge transport losses, and limits the performance of optoelectronic devices, and in particular those designed to harvest or detect photons.

Charge transport losses are typically described by the average distance that a photocarrier travels prior to its recombination event. The critical requirement for lossless charge transport is that the drift or diffusion distance (*L*_D_) must be longer than the active layer thickness (*d*). For inorganic crystalline semiconductors this distance is classically defined by the product of the charge carrier mobility and lifetime (*μτ*) regardless of whether the photocarrier driving force is the electric field (drift) or concentration gradient (diffusion)^1^. In strongly non-Langevin materials such as silicon and other inorganic crystalline semiconductors (where the recombination coefficient is typically > 10^5^ times lower compared to Langevin systems^2^), charges can pass each other at distances closer than Coulomb radius without recombination during transport. The reason for this is that the carrier mean free carrier path (the average distance between carrier collisions during random thermal motion) of ∼ 100 nm is much larger than the Coulomb radius (the distance at which the thermal energy equals the Coulomb energy) of ~ 5 nm.^3^ This implies that the mutual Coulombic attraction between positive and negative charges does not significantly affect the travel trajectory and the photocarrier lifetime represents the true nature of recombination. Therefore, the mobility-lifetime product can adequately describe the distance charges travel prior to recombination in these crystalline non-Langevin systems.

In contrast, in disordered organic semiconductors (an archetypal Langevin-system) the mean free path is defined by the carrier hopping distance (~ 1 nm), which is substantially shorter than the Coulomb radius (∼ 20 nm)^4^. Hence, when the charge carrier density is such that the average separation distance between charges is comparable to the Coulomb radius, charge carriers have a high probability of recombination because positive and negative charges are not able to escape their mutual Coulomb attraction. The recombination dynamics is then defined by the Langevin rate. The photocarrier lifetime under these conditions is strongly dependent upon the physical separation of negative and positive charges, which is determined by the carrier density, distribution, and, for example, on the formation of space charge regions^5^,^6^. Therefore, a single carrier lifetime cannot adequately characterize the entire device. In contrast the recombination coefficient is a material property, which is unaffected by the distribution of charge carriers. Futhermore, the photocarrier lifetime depends on its mobility^7^, which dictates the average velocity with which charges of opposite signs move with respect to each other. Given this dual dependency and the arguments above, the mobility-lifetime product (and hence *L*_D_) is clearly unsuitable as a universal “figure of merit” for the transport physics of organic semiconductors which are Langevin-type, as most are^4^.

Despite these considerations, the mobility-lifetime product is widely used as an appropriate predictive metric by which to assess and explain the performance of organic solar cell materials and architectures, and indeed more broadly photon-harvesting or detecting devices^8^,^9^,^10^,^11^,^12^,^13^. In this work, we address the fundamental processes determining the photoconductivity and charge transport losses in low mobility disordered films of organic semiconductors. We demonstrate that the classical mobility-lifetime approach is not a convenient parameter to describe the charge transport in these light harvesting systems. We independently measure the relevant charge transport parameters in operational devices, and directly relate these basic properties to the bimolecular recombination losses. The results show that the critical carrier density that triggers the onset of the recombination losses is determined by the charge density on the device electrodes. Based upon this physics we propose an alternative figure of merit allowing the minimization of charge transport losses in undoped disordered systems, where charge trapping does not dictate the photovoltaic performance. To this end, we employ intensity dependent PhotoCurrent (iPC) and Resistance dependent PhotoVoltage (RPV) measurements in two high efficiency organic solar cell (OSC) systems – each with quite different transport physics.

## Results

### Photovoltaic performance of solar cells

**Figure 1:**
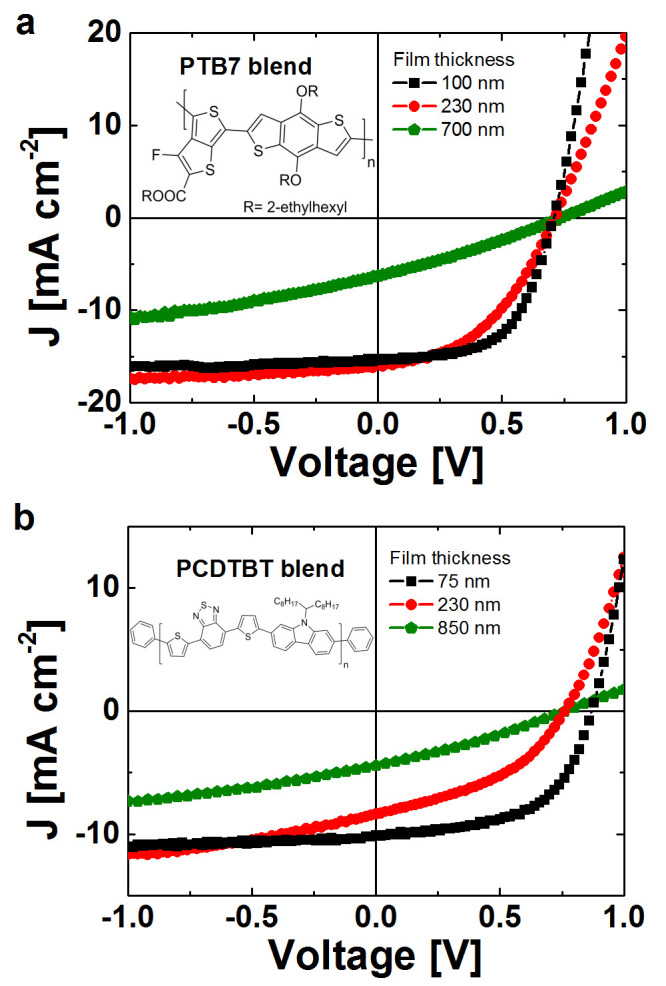
Average current density-voltage (*JV*) characteristics under standard AM 1.5G illumination of organic solar cells fabricated from (a) PTB7:PC70BM blends with 100 nm, 230 nm and 700 nm thick active layers and (b) PCDTBT:PC70BM blends with 75 nm, 230 nm and 850 nm thick junctions. The photovoltaic performance of the PCDTBT:PC70BM blends is much more susceptible to the film thickness of the active layer compared to PTB7:PC70BM.

The most efficient single junction organic solar cell architecture currently employed is the so-called bulk heterojunction (BHJ). This architecture has an active layer containing a blend of organic semiconductors with electron acceptor and electron donor characteristics. In our study we employed two high efficiency blends namely: poly[(4,8-bis{2-ethylhexyloxy}benzo[1,2-b:4,5-b′]dithiophene-2,6-diyl)(3-fluoro-2-{[2-ethylhexyl]carbonyl}thieno[3,4-b]thiophenediyl)]:[6,6]-phenyl-C_70_-butyric acid methyl ester (PTB7:PC70BM)^14^ and poly[*N*-9″-heptadecanyl-2,7-carbazole-*alt*-5,5-(4′,7′-di-2-thienyl-2′,1′,3′-benzothiadiazole)] (PCDTBT):PC70BM^15^. The structures of the two different polymers are provided Figure 1**.** These systems have been extensively studied^16^,^17^,^18^,^19^,^20^ and are known to have quite different charge transport properties (PTB7:PC70BM being superior). In this work we varied the junction thickness as an experimental parameter so as to access a range of key charge transport properties (recombination and mobility) in a systematic manner and study their impact upon device performance.

Figure 1 shows white light current density versus voltage (*JV*) plots obtained under standard AM 1.5G illumination for each polymer blend as a function of junction thickness. The plots are representative of fabrication batches containing multiple devices (see Methods). For PTB7:PC70BM solar cells shown in Figure 1 (a), the optimal junction thickness is 100 nm, and hence 230 nm and 700 nm are essentially sub-optimal and this is borne out by the *JV* curves. Similarly, Figure 1 (b) shows representative white light *JV* characteristics for the PCDTBT:PC70BM blend. In this case the optimal junction thickness is 75 nm – 230 nm and 850 nm being sub-optimal. The performance metrics including relevant statistics are summarized in Table 1. It has previously been demonstrated that the Fill Factor (FF) and PCE fall off rapidly for junctions > 80 nm for PCDTBT:PC70BM^18^, and we also observe the same trend. This has been attributed to poor charge transport in this blend, in particular low hole mobilities which leads to significant bimolecular recombination in thicker junctions under 1 sun operating conditions. The PTB7:PC70BM cells have considerably better charge transport and the PCE is maintained past 100 nm – although it does fall off for thicker junctions. Loss of FF still limits the optimal junction thickness to 100 nm–150 nm: increasing the thickness to 230 nm delivers higher short circuit current but FF losses lead to a reduced PCE of 5.1%. These observations are in-line with expectations.

### Photocarrier recombination losses

To quantify these transport losses and relate the observed losses in the photovoltaic performance in the non-optimal, thick active layer junctions to the losses in charge transport, we employed two techniques: iPC and RPV measurements. The iPC method has been extensively used to assess bimolecular losses in organic solar cells^17^,^21^,^22^,^23^,^24^,^25^ and relies upon the accurate measurement of photocurrent as a function of the input light intensity (typically from 1 to 100 mW cm^−2^). A linear fit to the photocurrent-intensity in a log-log plot is used (where the slope is often marked as *α*) to determine whether the device performance is limited by bimolecular recombination or not. Deviation from slope 1 shows the presence of significant bimolecular recombination. However, fitting a line to the photocurrent data over a narrow range of light intensities near this transition between the linear and the sub-linear regimes^22^,^26^,^27^ can result in an arbitrary slope and is prone to error. iPC measurements over a large range of light intensities are also crucial for characterizing organic photodiodes (OPDs) since the point of deviation determines the Linear Dynamic Range (LDR)–an important figure of merit in all photodetectors. Once again LDR measurements are often not sufficiently accurate to determine the deviation point^28^,^29^.

**Figure 2:**
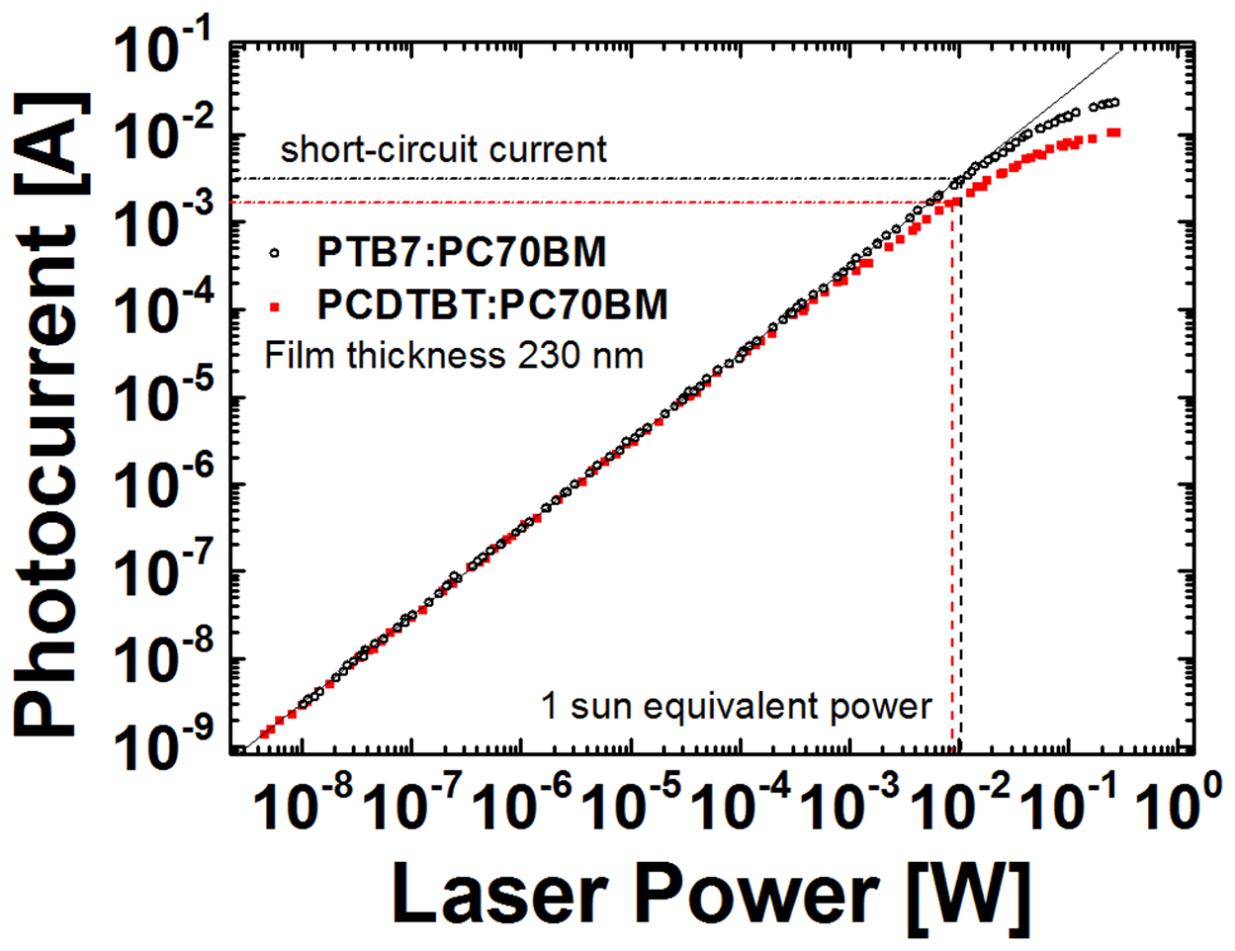
iPC results: the photocurrent measured as a function of the incident laser power varied by orders of magnitude in PTB7:PC70BM blends and PCDTBT:PC70BM blends with junction thickness of ∼ 230 nm. The 1 sun equivalent laser power is marked by the dashed lines. The bimolecular recombination losses appear at the highest laser powers when the photocurrent becomes non-linear.

With these considerations in mind, we have extended the measurement range by many orders of magnitude. Figure 2 shows extended range iPC results for both polymer blends with photocurrent measured between ~ 3 × 10^−9^ W and ~ 3 × 10^−1^ W of laser power (note, the illuminated device area is 0.2 cm^2^). The junctions in both cases were ~ 230 nm thick and the iPC measurements on other junction thicknesses are provided in the Supplementary Information for completeness (see Supplementary Fig. 1). We observe that the photocurrent increases linearly at low light intensities until a critical current that we call the deviation current is reached. Beyond the deviation current, the bimolecular recombination rate becomes comparable with the extraction rate and causes the photocurrent to deviate from linearity. In the linear regime the photocurrent is only affected by first order losses (i.e., those with a rate proportional to the first power of the illumination power). The origin of the first order losses has been attributed to a number of photophysical processes including incomplete absorption and geminate recombination^21^,^23^. Note, that a linear scaling of the photocurrent with the laser power does not guarantee the absence of photocarrier recombination in cases where there are a large amount of long-lived trap states or strong doping^17^,^30^,^31^,^32^. It has been, however, previously argued that first order photocarrier recombination (or trap-assisted recombination) is not relevant in optimized and efficient OSCs^32^,^33^. In particular, dominant second order recombination dynamics have been observed in optimized polymer:PC70BM blends^34^,^35^. In this work we have also experimentally confirmed the absence of long-lived trap-induced recombination losses by repetitive RPV shots in the optimized and the 230 nm thick junctions (Supplementary Note 1 and Supplementary Fig. 2), while dark-CELIV transients prove the absence of doping induced charges (Supplementary Fig. 3). Since first order recombination is not significant in the polymer-fullerene combinations of this work we focus on the impact of the charge transport parameters on the transition from the linear to the nonlinear iPC regime, which corresponds to the onset of substantial bimolecular recombination losses with a non-linear recombination order.

**Figure 3:**
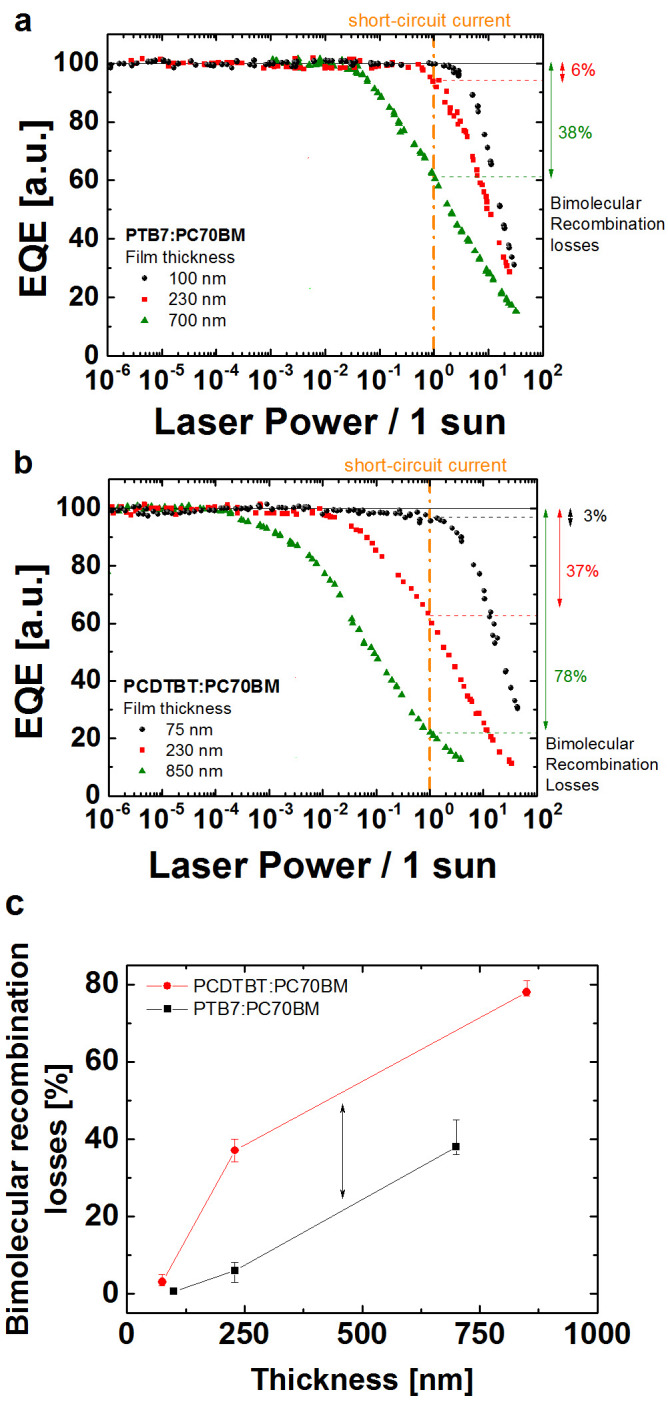
External Quantum Efficiencies (EQEs) (re-plotted from representations such as in Figure 2) shown as a function of the incident laser power for the different active layer thicknesses of (a) PTB7:PC70BM and (b) PCDTBT:PC70BM blends. The EQEs were normalized to 100% and the laser power to the 1 sun equivalent power to visualize the bimolecular recombination losses at the short-circuit conditions. This methodology allows one to quantify the photocarrier bimolecular recombination losses in actual solar cells under close-to-operational conditions. Figure (c) shows the recombination losses estimated from the Figures (a) and (b) and plotted as a function of the active layer thickness.

In order to better visualize the onset of photocarrier (bimolecular) recombination, in Figure 3 (a) and (b) we have re-plotted the iPC data as External Quantum Efficiency (EQE – the ratio of the photocurrent with light power) *versus* input light power. This process creates a non-logarithmic y-axis to visualize and compare more accurately the deviation points for all the junction thicknesses in both systems. We have also normalized the x-and-y-axes respectively to 1 sun equivalent power (i.e., the laser power at the short-circuit current) and the EQE in the constant regime to 100%. Note, this normalization sets the absorption and generation efficiency of the EQE to 100%. Therefore losses in the normalized EQE directly show losses in the transport (collection) efficiency. Figure 3 (a) shows the PTB7:PC70BM data and one observes that there are minimal recombination losses for the 100 nm and 230 nm thick junction solar cells up to 1 sun equivalent power (<1% and ~ 6% loss, respectively). However, in the 700 nm junction device, significant recombination losses are observed (~ 38%). For the PCDTBT:PC70BM devices, shown in Figure 3 (b), again only minor losses were observed in the highest efficiency, 75 nm thick junction cell. In contrast to the PTB7:PC70BM system, the 230 nm device displays considerable 1 sun recombination losses (transport efficiency reduced by ~ 37%). As the active layer is further increased to 850 nm, recombination decreases the transport efficiency substantially by ~ 78%. The recombination losses for all devices are summarized in Figure 3 (c). In both blend systems, the recombination losses are observed to follow the same trend as the solar cell performance metrics. It is worth noting that the trends in the two systems are similar, but with the effect of the recombination losses in the PTB7:PC70BM blends being shifted to thicker junctions.

### Origin of photocarrier bimolecular recombination losses

To further understand the losses in charge transport, we have measured the charge carrier mobilities and recombination coefficients in the devices. It should be noted that it was essential for this work to compare the mobility values (or transit times) with the recombination onset on the same devices. The well-known space charge limited current measurement technique was not applicable because the *J* ∼ *U*^2^ dependence of the Mott-Gurney law cannot be observed for the operational devices^20^ and mobilities obtained on pristine films are usually not the same as in blends of two organic semiconductors^16^. Therefore, we have used the RPV^19^,^20^ and High Intensity Resistance dependent PhotoVoltage (HI-RPV)^20^,^36^ techniques to determine the mobility of both electrons and holes as well as the bimolecular recombination coefficient ratio *β*_L_*/β* (where *β* is the actual and *β*_L_ the Langevin recombination coefficient) (Supplementary Fig. 4 to Supplementary Fig. 7). We found thickness independent dispersive carrier mobilities and bimolecular reduction factors: *μ*_electron_ ~ 3 × 10^−3^ cm^2^ V^−1^s^−1^, *μ*_hole_ ~ 3 × 10^−4^ cm^2^ V^−1^s^−1^ and *β*_L_*/β* ~ 50 in PTB7:PC70BM devices. The mobility of holes is ~ 10 times lower and the bimolecular recombination coefficient ratio is ~ 2 times lower in the PCDTBT:PC70BM devices, while a similar electron mobility was observed in both blends. An interesting observation is that the measured charge carrier mobility and *β*_L_*/β* are almost the same for all the studied film thicknesses for each polymer:PC70BM blend (Supplementary Fig. 5 and Supplementary Fig. 7). This suggests that the well controlled device preparation conditions did not result in any significant change in film structure that may have affected the charge transport.

Figure 3 also illustrates that the substantial bimolecular recombination losses appear at different photocarrier densities, which are dependent upon the junction thickness and blend system. Similarly, the space charge limited current (*I*_SCLC_) is determined by the density of space charge^5^. The *I*_SCLC_ has been shown to follow a square root dependence on the bimolecular recombination coefficient ratio^37^ (*β*_L_/*β*)^1/2^. It has also been previously demonstrated that the space charge limited photocurrent is proportional to the extraction rate of the slower charge carriers because they create a “bottleneck” for charge transport forming the space charge and causing the bimolecular recombination losses^23^,^38^. Therefore, the following expression can then be generalized:


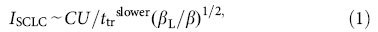
(Eq. 1) where *C* is the device capacitance, *U* is the effective voltage (superposition of built-in and external) and *t*_tr_^slower^ is the transit time of slower charge carrier species.

**Figure 4:**
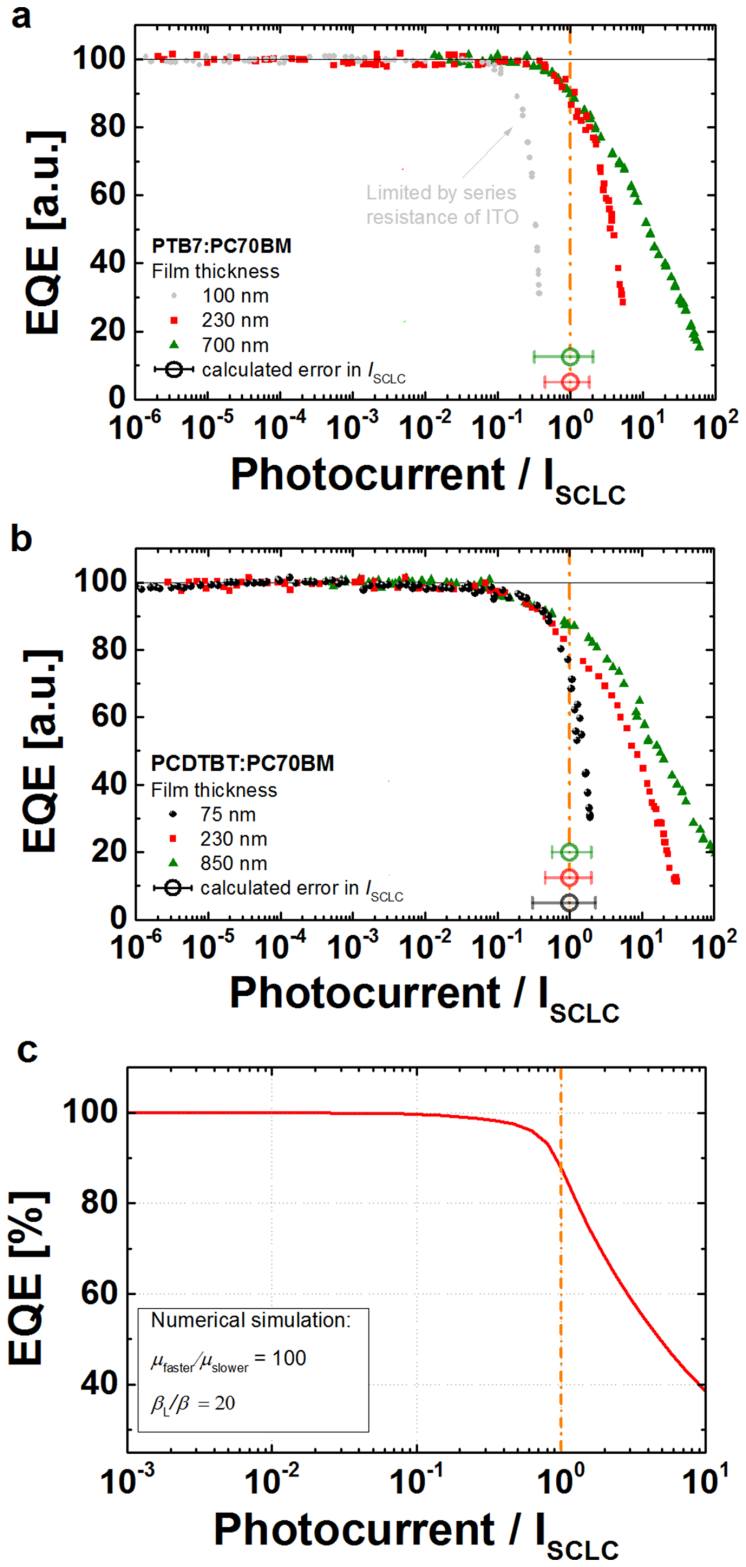
Normalized External Quantum Efficiencies (EQEs) shown as a function of the measured photocurrent for the different active layer thicknesses of (a) PTB7:PC70BM blends and (b) PCDTBT:PC70BM blends. The photocurrent is normalized to the space charge limited current (*I*_SCLC_), which is calculated from the measured charge transport parameters using Equation 1. When the actual measured photocurrent approaches the space charge limited current, substantial recombination losses manifest implying that the electrode defined space charge density controls the critical drift distance (*L*_D_ ∼ *d*, where *d* is the device thickness). (c) Numerically simulated EQEs as a function of the photocurrent confirm that the deviation is caused by the *I*_SCLC_, where the appearance of the first recombination losses can occur at a slightly lower photocurrent compared to *I*_SCLC_.

Using the measured slower carrier mobilities/transit times and recombination coefficients we can calculate the *I*_SCLC_ for each device (see Methods) and replot the previous EQEs (Figure 3) as a function of the photocurrent normalized to the *I*_SCLC _for the PTB7:PC70BM and PCDTBT:PC70BM blends in Figure 4 (a) and (b), respectively. Note, that the calculated *I*_SCLC_ values vary over many orders of magnitude mainly because of differences in the slower carrier transit times due to the different junction thicknesses.

The key observation from Figure 4 is that the bimolecular recombination losses start when the photocurrent reaches approximately the *I*_SCLC_ value, regardless of the active layer thickness. This implies that the critical charge carrier density that causes significant bimolecular recombination (compared to the extraction rate) is approximately equal to the surface charge density stored on the electrodes (*CU*), while the recombination coefficient ratio allows this critical density to be larger. The results are also confirmed in photodetectors with the same device architectures using applied external voltages to facilitate the charge transport and extraction. The bimolecular recombination losses are typically smaller at higher applied reverse biases, because the applied voltage increases the charge carrier drift velocity and the value of *CU* (Supplementary Figure 8 (a)). Nevertheless, even as the applied bias voltage is varied, the onset of substantial losses continues to coincide with the *I*_SCLC_ (Supplementary Figure 8 (b)). When a forward bias is applied (relevant to solar cells at operational conditions) the recombination losses increase (Supplementary Fig. 9).

Numerical EQE simulations, shown in Figure 4 (c) for a system with a mobility ratio of 100 and a recombination coefficient ratio of 20 further confirm the validity of Equation 1, the role of the *μ*_s _(*β*_L_/*β)*^1/2^ product and the space charge current limit. Moreover, these simulations can be used to predict the onset of bimolecular recombination losses as a function of experimental conditions such as the impact of the mobility ratio, recombination coefficient, the series resistance and the light absorption profile (see Supplementary Note 2 and 3; Supplementary Fig. 10 to Supplementary Fig. 12).

## Discussion

### Space charge determined photocarrier drift distance

**Figure 5:**
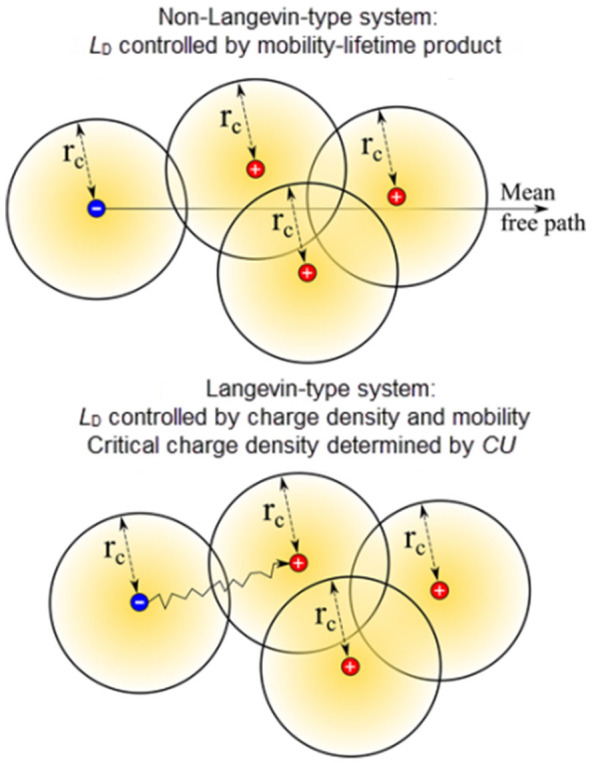
Schematic drawing showing the nature of charge carrier transport in non-Langevin (a) and Langevin-type systems (b). The photocarrier drift distance (*L*_D_) in non-Langevin systems is adequately described by the mobility-lifetime product because in these typically highly ordered systems the photocarrier mean free path is much larger that the Coulomb radius (*r*_c_). The photocarrier drift distance in Langevin-type systems is determined by the physical separation between the charges and their mobility. The critical charge density that triggers significant recombination (compared to the extraction rate) is determined by the electrode charge density *CU*. This situation is relevant to disordered structures where the photocarrier hopping distance is much smaller that the Coulomb radius (localized charge transport).

Drawing these experimental results together demonstrates that significant bimolecular recombination losses appear at very specific light intensities, junction thicknesses and applied voltages, depending upon the materials system in question. The experimental results suggest, that when the photocurrent matches the space charge limited current (i.e., when the photocarrier density is close to the *CU* space charge defined by the electrodes in Langevin-type systems), then the photocarrier drift distance becomes comparable to the junction thickness (*L*_D_ ∼ *d*) and substantial recombination losses emerge. Referring back to the Introduction, in which we compared the charge carrier drift distances in two classes of materials, non-Langevin and Langevin, we reiterate that in the latter the critical photocarrier lifetime and drift distance are dependent upon carrier density. The density is defined by a number of material and device related parameters such as the light intensity, optical cavity effects, quantum efficiency of charge generation, film thickness, the photocarrier mobility, and others. This, in addition to the observed space charge dependent drift distance, clarifies that the *µτ* product (and therefore the drift distance itself) is not an independent intrinsic parameter that can be conveniently used as a comparative figure of merit to understand the charge transport physics. Importantly, the *µτ* product can also not be used to determine the critical active layer thickness to minimize the bimolecular recombination losses. These concepts and results are visualized in Figure 5.

Based upon these considerations we propose the product of the materials parameters *μ*_s _(*β*_L_/*β)*^1/2^ from Equation 1 as a comparative transport figure of merit because it determines the decisive *I*_SCLC_. It is important to note, however, that this figure of merit alone is not sufficient for describing the performance of the actual devices, because the recombination losses are governed by additional device related parameters, such as the film thickness, dielectric constant (both of which define the device capacitance) and effective voltage. Figure 4 shows that significant bimolecular recombination losses can be avoided only when the *I*_SCLC_ is greater than the actual photocurrent produced by the solar cell (see SI Supplementary Fig. 13 for the minimum *μ*_s _(*β*_L_/*β)*^1/2^ required to minimize the bimolecular recombination for a given active layer thickness and achievable photocurrent).

Finally, we note the influence of the transport and recombination dynamics in our two studied systems: the observed differences in the junction thickness dependent recombination losses are explained by the 10 times higher value of the slower carrier mobility and the ∼ 2 times higher bimolecular recombination reduction factor in the PTB7 blends as compared to the PCDTBT blends. This allows the PTB7 devices to work efficiently with slightly thicker junctions (∼ 230 nm). Our results also demonstrate the performance benefit due to the suppressed non-Langevin bimolecular recombination rate in all our devices (~ 50 times in PTB7 blends and ~ 25 times in PCDTBT blends) (see Supplementary Fig. 14). Therefore, improving the carrier mobility is not the only transport strategy to deliver higher overall PCEs. In summary, increasing the *μ*_s _(*β*_L_/*β)*^1/2^ product allows: (a) the device to operate efficiently at a higher maximum power point *V*_mp_ (increasing the FF), because a lower effective voltage is sufficient to extract the carriers without significant recombination losses; (b) the short-circuit current density (*J*_SC_) to be increased if the system is limited by bimolecular recombination at the short-circuit condition; and (c) an increase of *V*_oc_ *via* an enhanced carrier concentration^39^,^40^. This means that thicker junctions can be used to improve the efficiency of light harvesting systems.

## Conclusions

We have clarified that the conventional figure of merit (the *µτ* product or the drift distance *L*_D_) is not appropriate for a comparative analysis of charge transport losses in organic solar cells due to the photocarrier mobility and density dependent lifetime. It is argued that this is generally the case for a broad range of high performance light harvesting systems made of disordered low mobility and undoped materials. We found that the electrode charge density marks the onset of significant bimolecular recombination losses and therefore controls the critical photocarrier drift distance (*L*_D_ ∼ *d*). Based upon this physics we propose a new figure of merit for material and device characterization – the mobility-recombination-coefficient product *μ*_s _(*β*_L_/*β)*^1/2^. This parameter allows to minimize photocarrier recombination losses and to maximize the photovoltaic performance of organic solar cells and photodetectors. We verify this analysis in our model systems and find that the PTB7:PC70BM blends are superior compared to PCDTBT:PC70BM blends from a charge transport perspective because of the higher hole mobility and stronger suppressed recombination. Our work establishes a set of design rules to allow thicker junctions in organic solar cells whilst maintaining a high fill factor and power conversion efficiency. This is advantageous from a manufacturing perspective and offers an approach to improve the light harvesting efficiency of photovoltaic and photodetecting devices fabricated from low mobility materials.

## Methods

### Device preparation

The substrates (PEDOT:PSS/ITO/glass) were prepared as described in ref. 41 and the active layer (junction) solution of PTB7 (purchased from 1-Material, 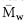 = 97.5 kDa, PDI = 2.1) and PC70BM (American Dye Source, Inc., Canada) was fabricated by using a 1:1.5 blend ratio by weight in chlorobenzene (CB) with 3% 1,8-diiodoctane (DIO) by volume. Solar cells with three different junction thicknesses were prepared by using a total concentration of 31 mg/cm^3^ for the 100 nm and 230 nm thick blends respectively, while a concentration of 45 mg/cm^3^ was used to fabricate the 700 nm thick blend. The solutions were spun cast at 2200 rpm, 400 rpm and 600 rpm for 120 s, respectively. The films were subsequently dried at 70°C. The active layer solution of PCDTBT (SJPC, Canada, 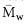 = 122 200 g/mol, PDI = 5.4) and PC70BM was prepared by using a 1:4 blend ratio by weight in 1,2-dichlorobenzene (DCB) following the procedure described ref. 42. Solar cells with three active layer thicknesses, 75 nm, 230 nm and 850 nm were fabricated by using a total concentration 25 mg/cm^3^ for the 100 nm and 230 nm thick blends respectively, while a concentration of 40 mg/cm^3^ was used to fabricate the 850 nm thick film. The solutions were spun cast at 2000 rpm, 500 rpm and 500 rpm for 90 s, respectively. The active layer thicknesses were measured with a DekTak 150 profilometer. All devices were completed by vacuum evaporation of 1.2 nm of samarium followed by 75 nm of aluminum under a 10^−6^ mbar vacuum. The device area was 0.2 cm^2^ for *JV*, iPC and EQE measurements, and a 3.5mm^2 ^for the RPV measurements, respectively. Note, we found the RPV measurement results were independent of the area of the pixel. All device fabrication took place within a glove box with <1 ppm O_2_ and H_2_O and *JV* and EQE measurements were also performed inside a glove box. Subsequently the devices were encapsulated for the iPC measurements.

### Current density-voltage characteristics

*JV* curves were obtained in a 2-wire source sense configuration and an illumination mask was used to prevent photocurrent collection from outside of the active area. The presented PCEs correspond to average values of 6 pixels after several *JV*-measurements and represent the efficiencies of the devices directly before the iPC measurements were conducted. An Abet Class AAA solar simulator was used as the illumination source providing ~ 100 mW cm^−2^ of AM1.5G light. The exact illumination intensity was used for efficiency calculations and the simulator was calibrated with a standards traceable NREL photodiode.

### Light intensity dependent measurements

iPC measurements were performed with a 532 nm continuous wave laser (Ningbo Lasever Inc.) providing a power of 1 W. Optical filters (ThorLabs) were used to attenuate the laser power and the photocurrent transients were recorded with an Agilent semiconductor device analyser (B1500A). Each measured data point corresponded to a steady state photocurrent measurement of the OSC at the respective incident laser power, which was simultaneously measured with a Silicon photodetector to improve the accuracy of the measurement. The error bars in Figure 3 (c) were estimated from the spread of the EQE values at the 1 sun equivalent power and the uncertainty in the short-circuit current. The error analysis for the calculated *I*_SCLC_ was conducted as follows: The circles in Figure 4 represent the calculated *I*_SCLC_ from the actual measured charge transport parameters on duplicate devices. In particular, the mean slower carrier transit time (Supplementary Figure 4) was used and the built-in voltage (*U_BI_*) approximated by *V*_oc_. The values of the *I*_SCLC_ are 78.3 mA, 4.4 mA, 0.17 mA for the 100 nm, 230 nm and the 700 nm thick PTB7:PC70BM junctions, and 15.1 mA, 0.34 mA, 0.01 mA for the 75 nm, 230 nm and the 850 nm thick PCDTBT:PC70BM junctions. For the upper error bar a 10% thicker active layer was assumed, a *U*_BI_ that is 0.05V higher than *V*_oc_, *β*_L_/*β* = *β*_L_/*β* + 5, and for *t*_tr_^slower^ the lower limit of the dispersive slower carrier transit time range (Supplementary Figure 4) was taken. For the lower error bar a 10% thinner active layer was assumed, *V*_oc_ as the built-in voltage, *β*_L_/*β* = *β*_L_/*β* −5 and the transit time of the slowest carriers in the device. Note, that the range of the error bar is mainly determined by the measured dispersive slower carrier mobility range, while the upper error bar represents a rather unrealistic case for the *I*_SCLC_, because that would imply that the fastest of the slower carriers determine the onset of the bimolecular recombination losses.

### Mobility, recombination coefficient, trapping and dark-CELIV measurements

RPV transients for mobility, *β*_L_*/β* and charge trapping measurements were recorded with an oscilloscope (LeCroy WaveRunner 6200A) with different external load resistances (*R*_Load_), while a delay generator (Stanford Research Systems DG535) was used to trigger a function generator (Agilent 33250A) and a pulsed Nd:Yag laser (Brio Quantel) with a pulse length of 10 ns. An excitation wavelength of 532 nm was used to generate the charge carriers, while neutral optical density (OD) filters were used to attenuate the ~ 50 mJ energy output. The RPV transients were measured under various applied biases. Low laser pulse intensities (∼ OD 7) were used for the RPV mobility measurements to avoid space charge effects^19^. In contrast a high laser intensity (OD 3.5) was used to measure the bimolecular recombination coefficient on the same films. CELIV transients were recorded in the dark with the same experimental setup.

### Numerical simulations

The numerical simulations implement the key processes that occur in organic solar cells, such as carrier drift, diffusion, trapping, non-geminate recombination and space charge effects by taking into account the circuit resistance and the influence of the light absorption profile. Details of this model can be found in the Supplementary Information Methods.

## Author Contributions

M.S. fabricated the devices and performed the measurements, and A.P. and M.S. analysed and interpreted the data. A.A. developed the experimental setup and A.P. conceptualized the study. B.P. wrote the software and performed the simulations. P.L.B. and P.M. supervised the experimental study. R.D.W. supervised the theoretical study and assisted with developing the software and simulations. G.J. provided fundamental insight and critique. A.A. and B.P. equally contributed to performance of the research and its outcomes, and all authors contributed to the development and writing of the manuscript which was drafted by M.S. and A.P.

## Supplementary Material

Supplementary Information Supplementary Information

## Figures and Tables

**Table 1:** Device performance parameters including standard errors of the studied PTB7:PC70BM and PCDTBT:PC70BM devices

Photovoltaic performance parameters						
Polymer blended with PC70BM and junction thickness	PTB7 100 nm	PTB7 230 nm	PTB7 700 nm	PCDTBT 75 nm	PCDTBT 230 nm	PCDTBT 850 nm
*J*_SC_ [mA/cm^2^]	15.3 (±0.3)	16.0 (±0.4)	6.3 (±1.7)	10.3 (±0.3)	8.3 (±0.8)	4.4 (±1.2)
*V*_OC_ [V]	0.7	0.71	0.72	0.84	0.75	0.76
FF [%]	59	45	28	56	42	33
PCE [%]	**6.3** (±0.1)	**5.1** (±0.2)	**1.3** (±0.4)	**4.8** (±0.1)	**2.6** (±0.2)	**1.1** (±0.3)
