# Supplementary material for: Photocarrier drift distance in organic solar cells and photodetectors
Source: Sci Rep. 2015 Apr 28;5:9949. doi: 10.1038/srep09949 (PMC4412075; doi:10.1038/srep09949)
Supplement: Supplementary Information [file srep09949-s1.docx]

# Supplementary Information

**Photocarrier drift distance in organic solar cells and photodetectors**

Martin Stolterfoht,^1^ Ardalan Armin,^1^ Bronson Philippa,^2^ Ronald D. White_,_^2^ Paul L. Burn,^1^ Paul Meredith,^1^ Gytis Juška^3^ and Almantas Pivrikas^1,*^

^1^Centre For Organic Photonics & Electronics (COPE), School of Chemistry and Molecular Biosciences and School of Mathematics and Physics, The University of Queensland, Brisbane 4072, Australia.

^2^School of Engineering and Physical Sciences, James Cook University, Townsville 4811, Australia

^3^Department of Solid State Electronics Vilnius University 10222 Vilnius, Lithuania

***almantas.pivrikas@uq.edu.au**

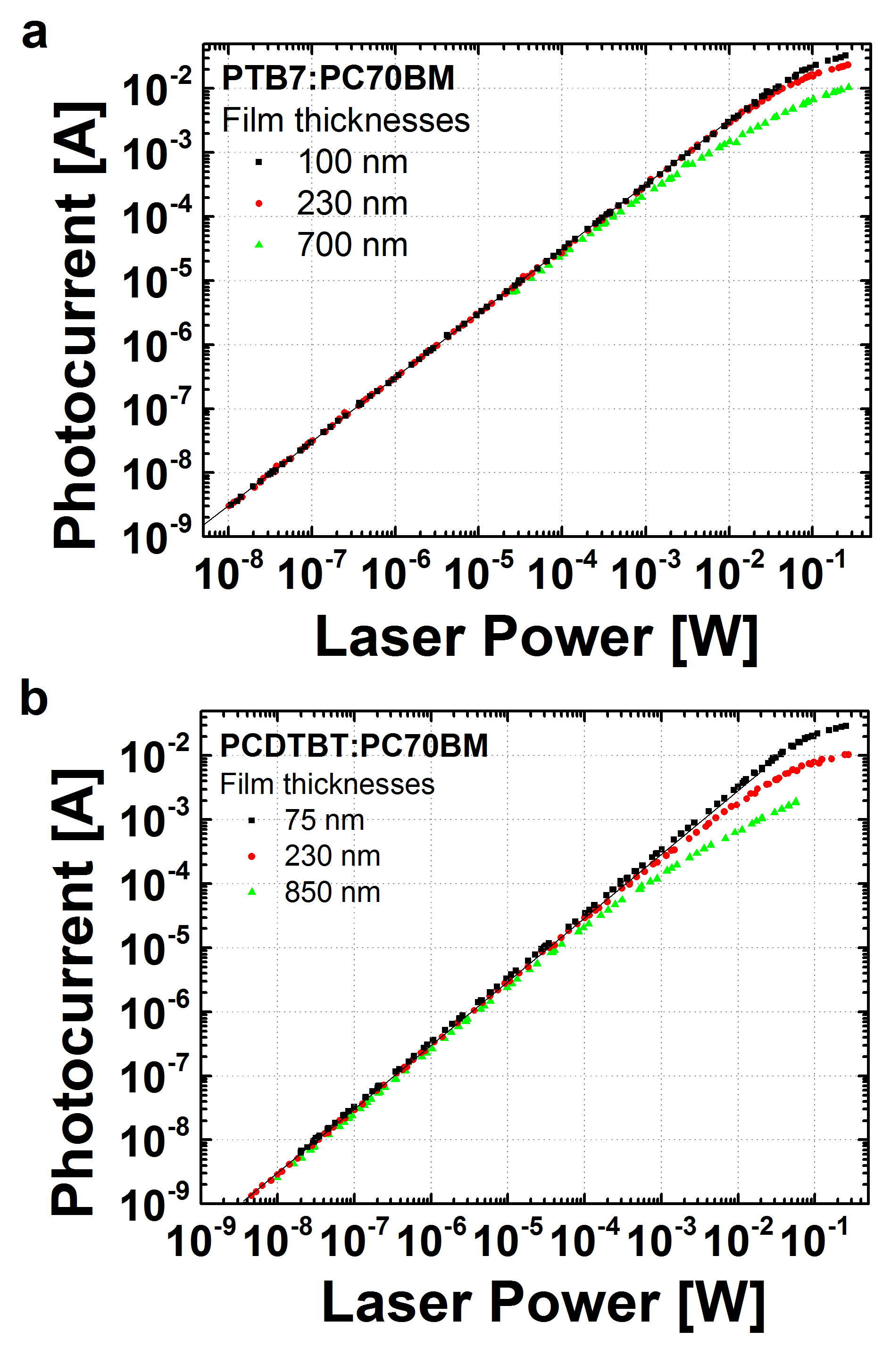


**Supplementary Figure 1.** Original iPC data measured in the studied devices and analysed in the main text.

**Supplementary Note 1.** Charge carrier trapping complicates the result interpretation of the iPC experiment. To check the presence of long-lived trap-induced recombination losses that can cause a first order recombination in the linear regime of iPC experiment, repetitive Resistance dependent PhotoVoltage (RPV) transients were measured [1, 2] (**Supplementary Figure 2**).


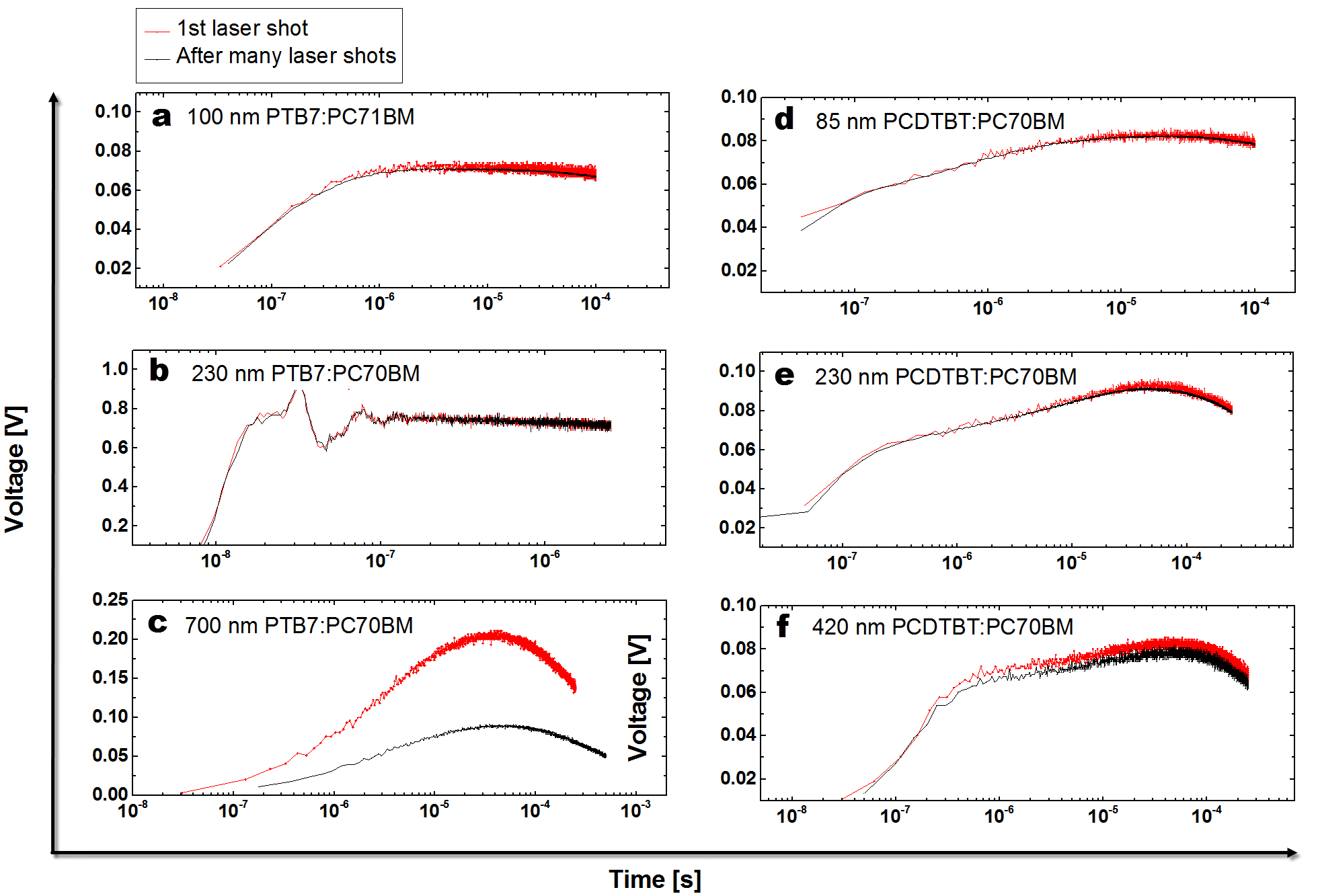


**Supplementary Figure 2.** Photovoltage transients measured to detect trap-induced recombination losses in the studied devices [1, 2]. The transient of the “first laser shot” (red) is recorded after allowing the devices to rest overnight, which allows deeply trapped charges to dissipate. The difference in the magnitude between the “first transient” and the transient after many laser shots (black) measured with a laser repetition rate of 20 Hz reveals if recombination losses are present due to long-lived (> 50ms) traps. Identical RPV transients in the efficient devices (a,b,d,e) indicate the absence of trap-induced recombination losses that can cause a significant first order recombination in the iPC experiment. Note, trap-induced recombination losses are observed in the thickest PTB7 device (c). However, this does not hinder the interpretation of the iPC experiments because a majority of the short-circuit current losses are determined by the bimolecular recombination with a non-linear recombination order. Also, the good agreement between the recombination onset and the *I*_SCLC_ (**Figure 4** main text) indicates no significant 1st order recombination losses in all studied devices.


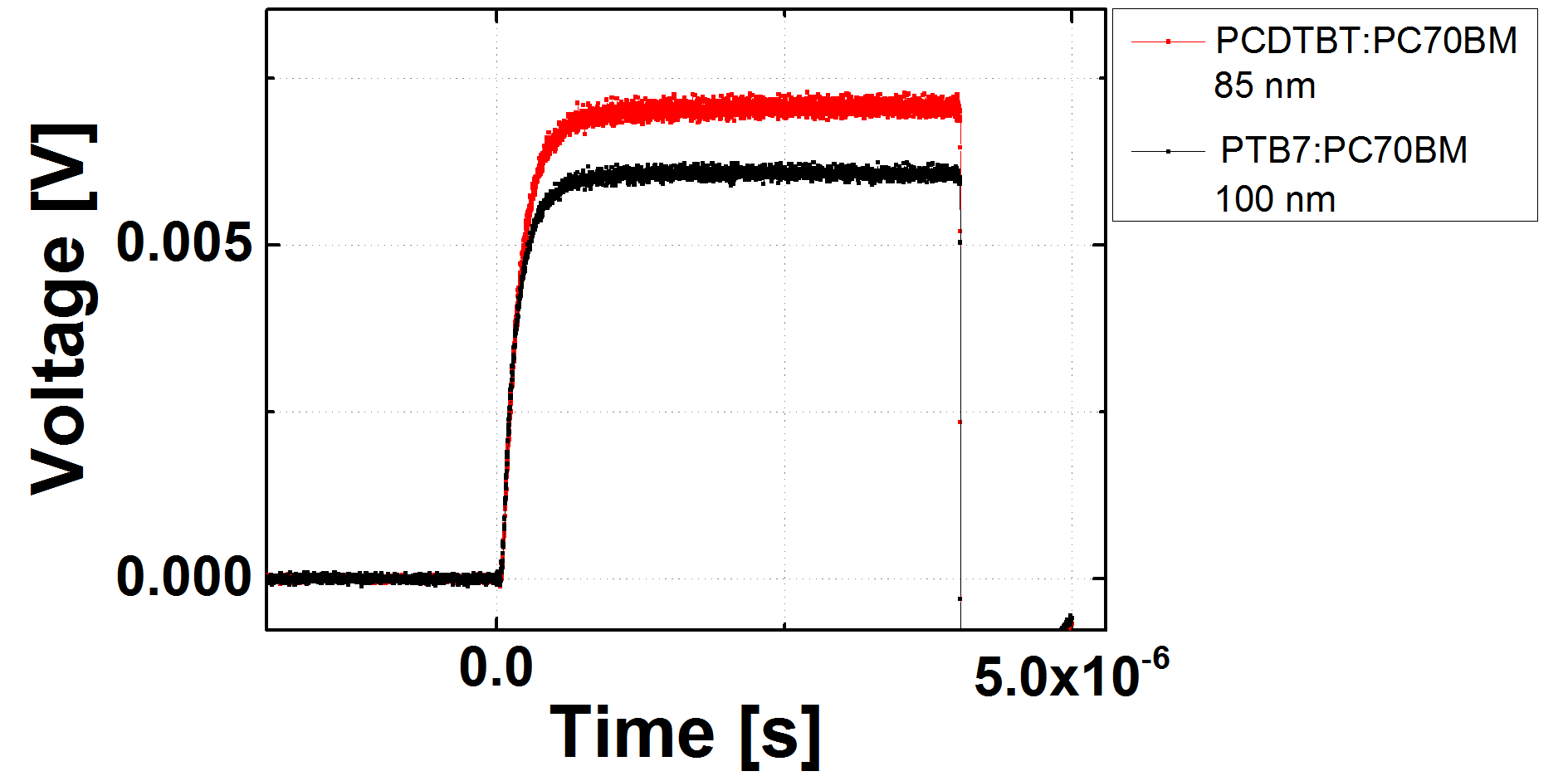
**Supplementary Figure 3.** Transient signals of the dark Charge Extraction by Linearly Increasing Voltage (dark-CELIV) experiment. The typically observed “flat” responses in the dark-CELIV transients in our studied polymer:PC70BM devices demonstrates the absence of doping induced equilibrium charge carriers [3]. This observation is used to confirm the absence of first order recombination losses in the linear iPC regime. The transients were recorded by using a load resistance of 20 Ω and a peak voltage of the triangular voltage pulse of 1 V.

**
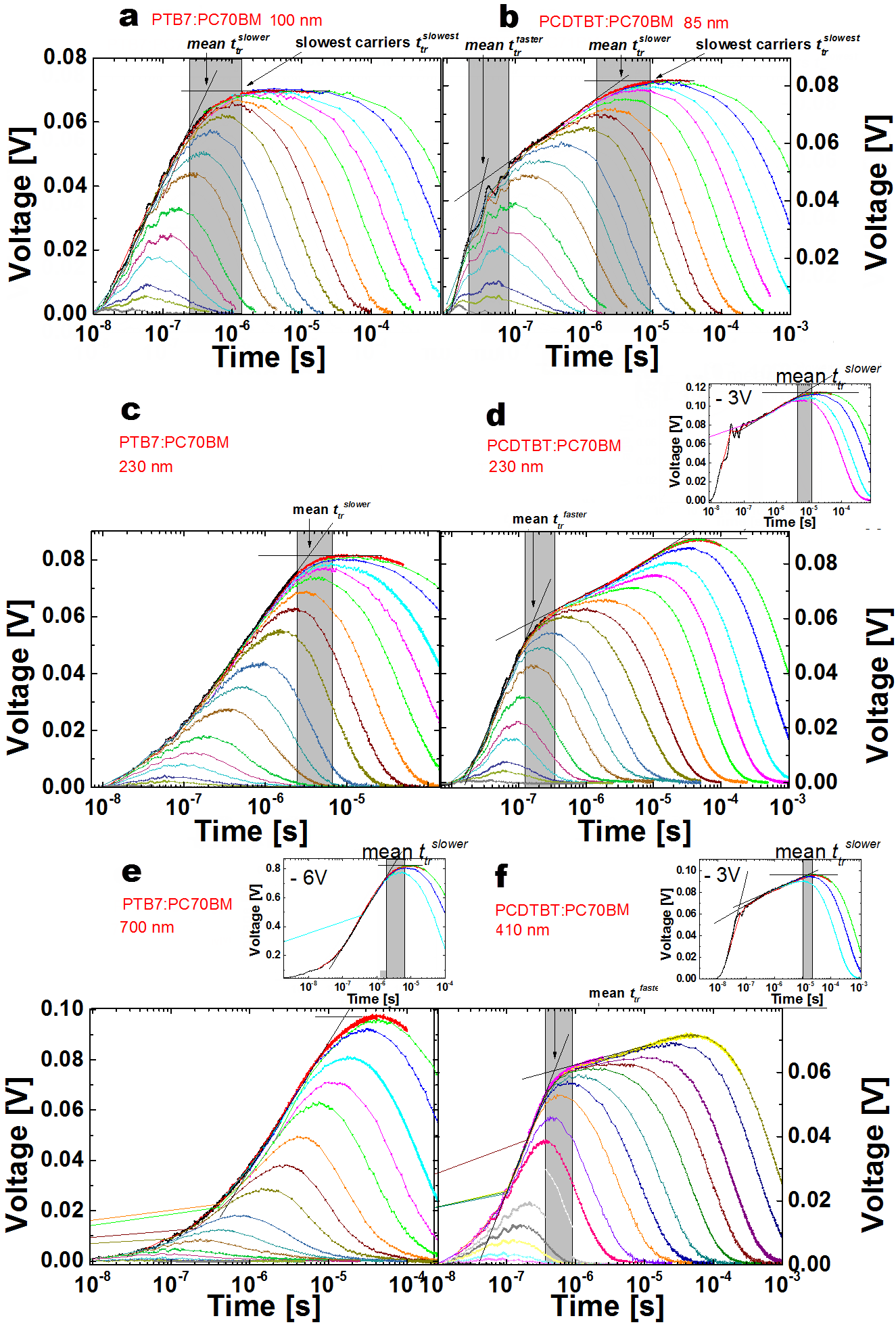
**

**Supplementary Figure 4.** Experimentally measured Resistance dependent PhotoVoltage (RPV) transient signals in the studied solar cells fabricated from PTB7:PC70BM blends (a,c,e) and PCDTBT:PC70BM blends (b,d,f). The details of the technique are described in [1]. The observed characteristic shoulders in the transients appear when all photogenerated electrons and/or holes are extracted from the film. Therefore, these shoulders represent the charge carrier transit times (*t*_tr_) from which the carrier mobilities are calculated. The mean electron (faster) and hole (slower) transit times are marked, from which the respective mean mobilities are estimated. A shift to longer transit times with increasing thickness is observed. The bigger panels correspond to measurements under zero bias conditions. In the case of thicker devices, it is necessary to apply a reverse bias to saturate the maximum of the photovoltage to correctly estimate the slower carrier transit time (see smaller panels in d,e,f). We note, that also the independence of the mobility on the applied voltage has been reported in the two studied polymer:PC70BM systems [1].


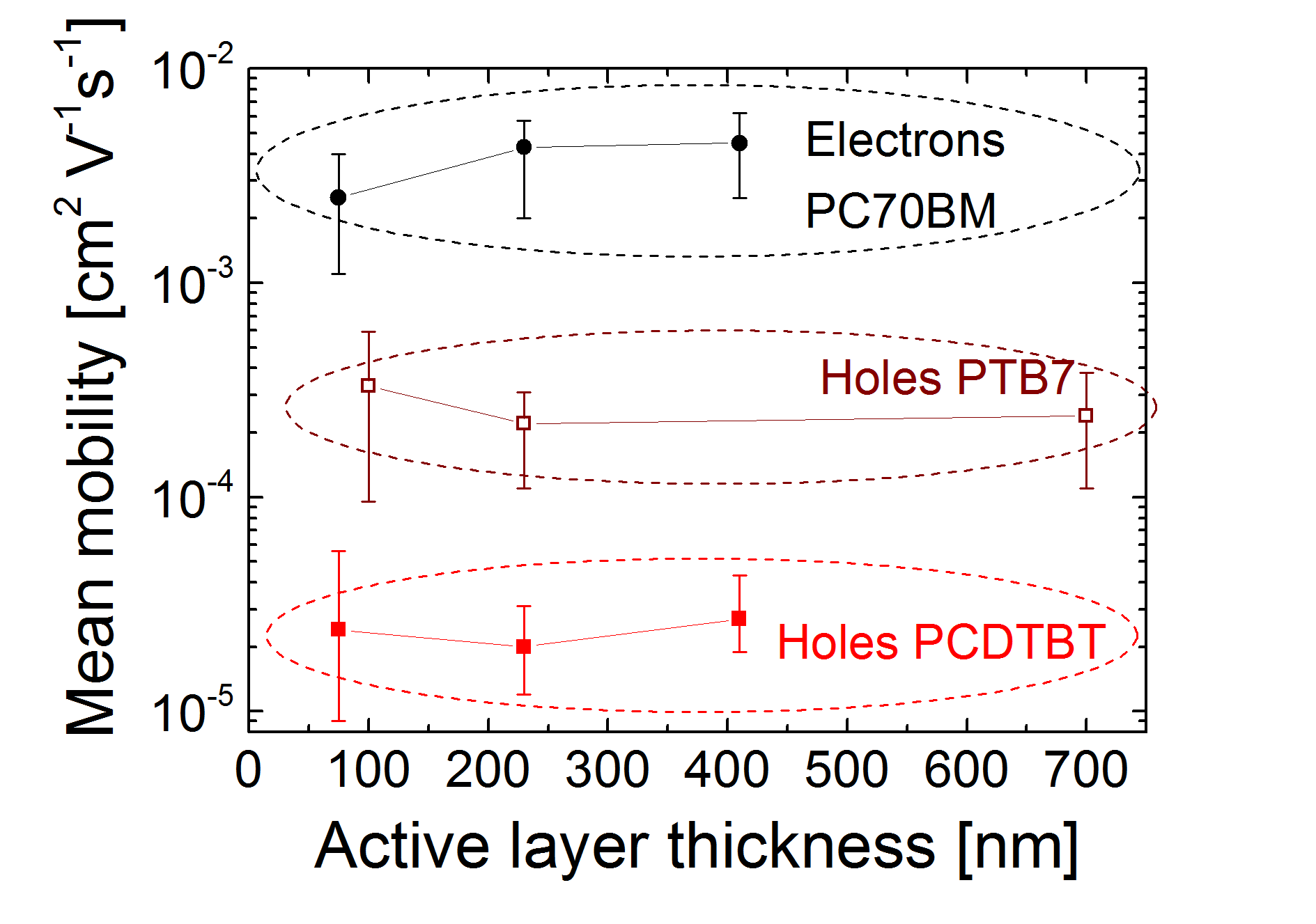


**Supplementary Figure 5.** The mean carrier mobilities with dispersion ranges obtained from the photovoltage transients in **Supplementary Figure 4** plotted as a function of the active layer thickness (*d*) of the studied devices**.** The mobility values were calculated from *μ* = *d*^2^ *t*_tr_^-1^ *U*^-1^, where *t*_tr_ was taken from the observed transit times (**Supplementary Figure 4**) and the voltage *U* was approximated as *V*_OC_.


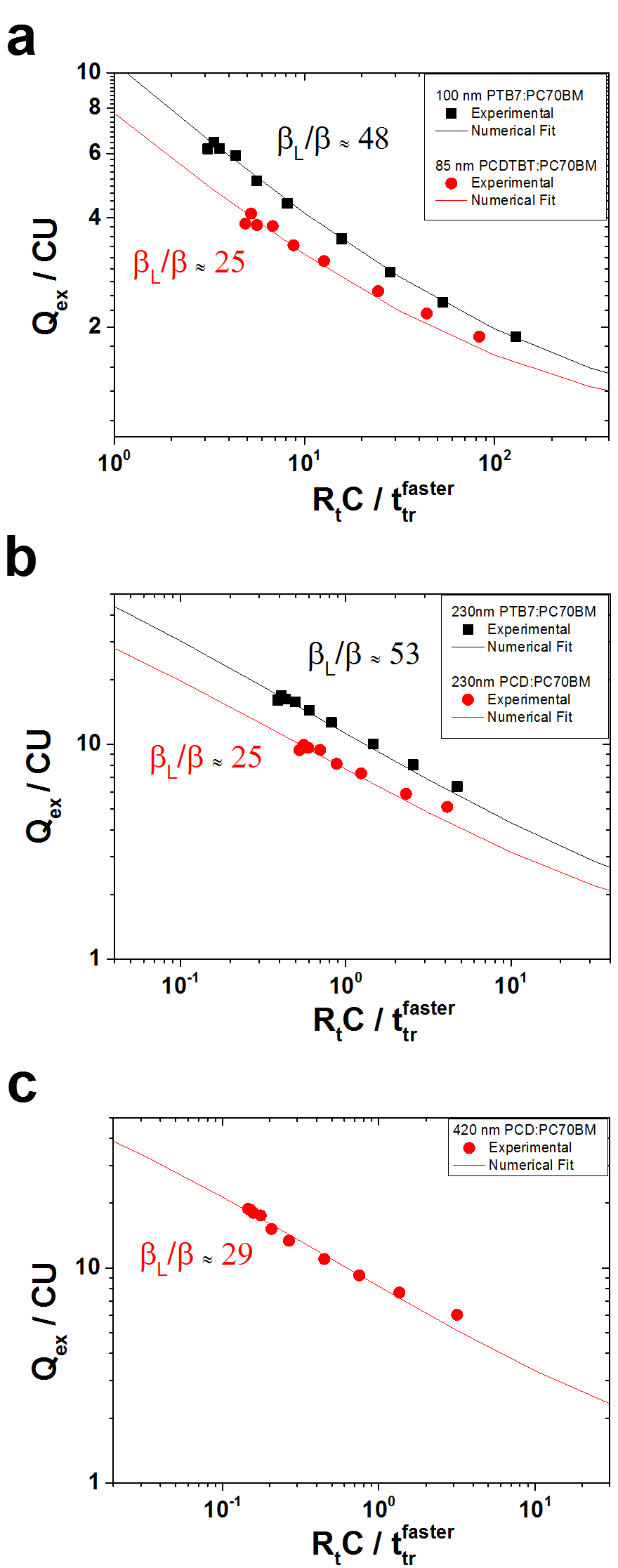


**Supplementary Figure 6.** The extracted charge *Q*_ex_ (normalized to the charge on the electrodes, *CU*) measured at high laser intensities as a function of the total circuit time constant *R*_t_*C* (normalized to the faster carrier transit time) for optimum devices: (a), the 230 nm thick devices; (b) and the thickest measured PCDTBT:PC70BM blend (c). By matching the experimental data points with numerical simulations the Langevin recombination reduction factor (*β*_L_*/β*) is directly estimated according to the technique recently introduced in [4]. In this experiment, the extracted charge (*Q*_ex_) is obtained by integrating the photovoltage transients (similar to the transients shown in **Supplementary Figure 4)**, but measured at very high laser intensities (such that the magnitude of the photovoltage produced by the device under test is saturated).


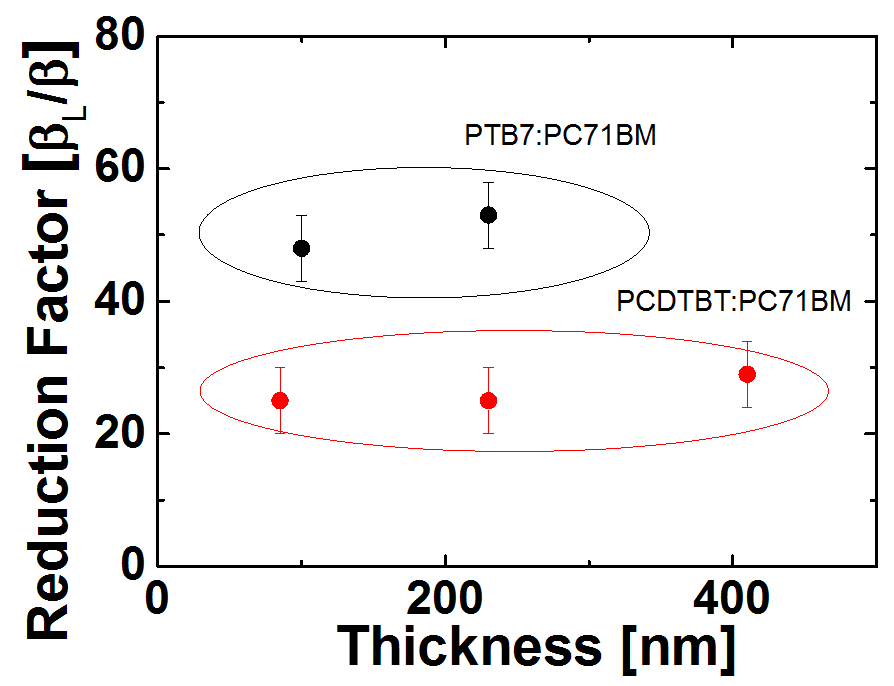


**Supplementary Figure 7.** The reduction factor of the Langevin recombination coefficient *β*_L_ obtained from **Supplementary Figure 6** plotted as a function of the active layer thickness of the studied devices. Almost identical recombination coefficients are observed regardless of the active layer thickness.


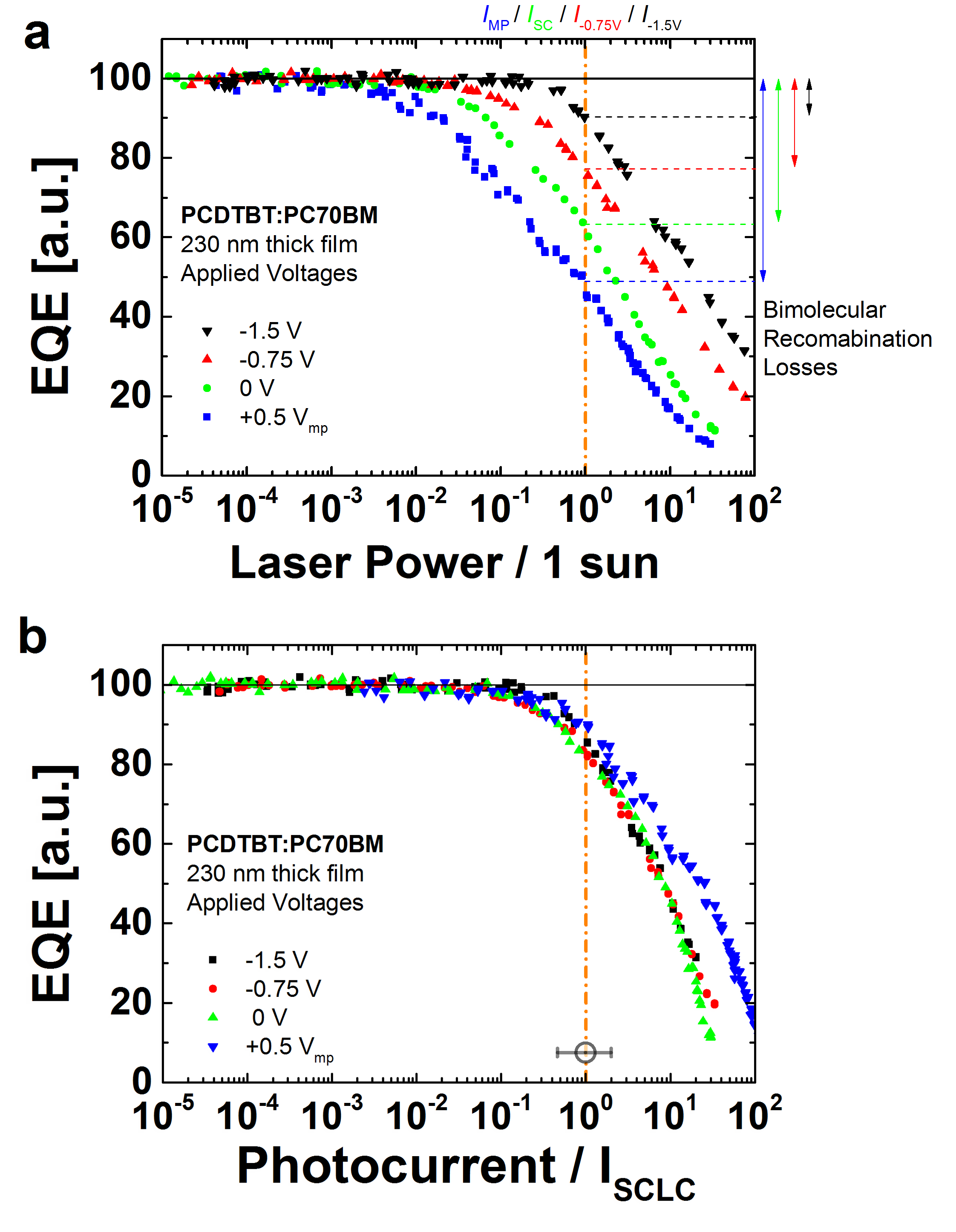


**Supplementary Figure 8. (a)** The experimentally measured EQEs normalized to 100% as a function of the laser power normalized to the 1 sun equivalent power at different applied voltages in photodetectors made of PCDTBT:PC70BM blends with active layer thickness of 230 nm. The bimolecular recombination losses are found to be smaller at higher applied reverse biases and likewise higher in forward bias. Typically an applied reverse bias in photodetectors facilitates the charge photogeneration and extraction, thereby increasing the linear dynamic range and detectivity of the devices. (b) The same EQEs as a function of the photocurrent normalized to the space charge limited current *I*_SCLC._ A very similar recombination onset can be observed regardless of the applied voltage. Note, that the EQE under forward bias +0.5 V is slightly larger than the EQEs at other applied voltages. However, this falls into the uncertainty of the *I*_SCLC_ determination to which the horizontal axis has been normalized (see Methods for an error analysis of the *I*_SCLC_). The results suggest that the voltage dependence of the bimolecular recombination onset is correctly described by Equation 1. Note, that onset of the bimolecular recombination is increased with the square of the effective voltage *U*_eff_ *^2^* because also the transit time depends on the effective voltage (~ 1*/U*_eff_) (as observed in [1]).


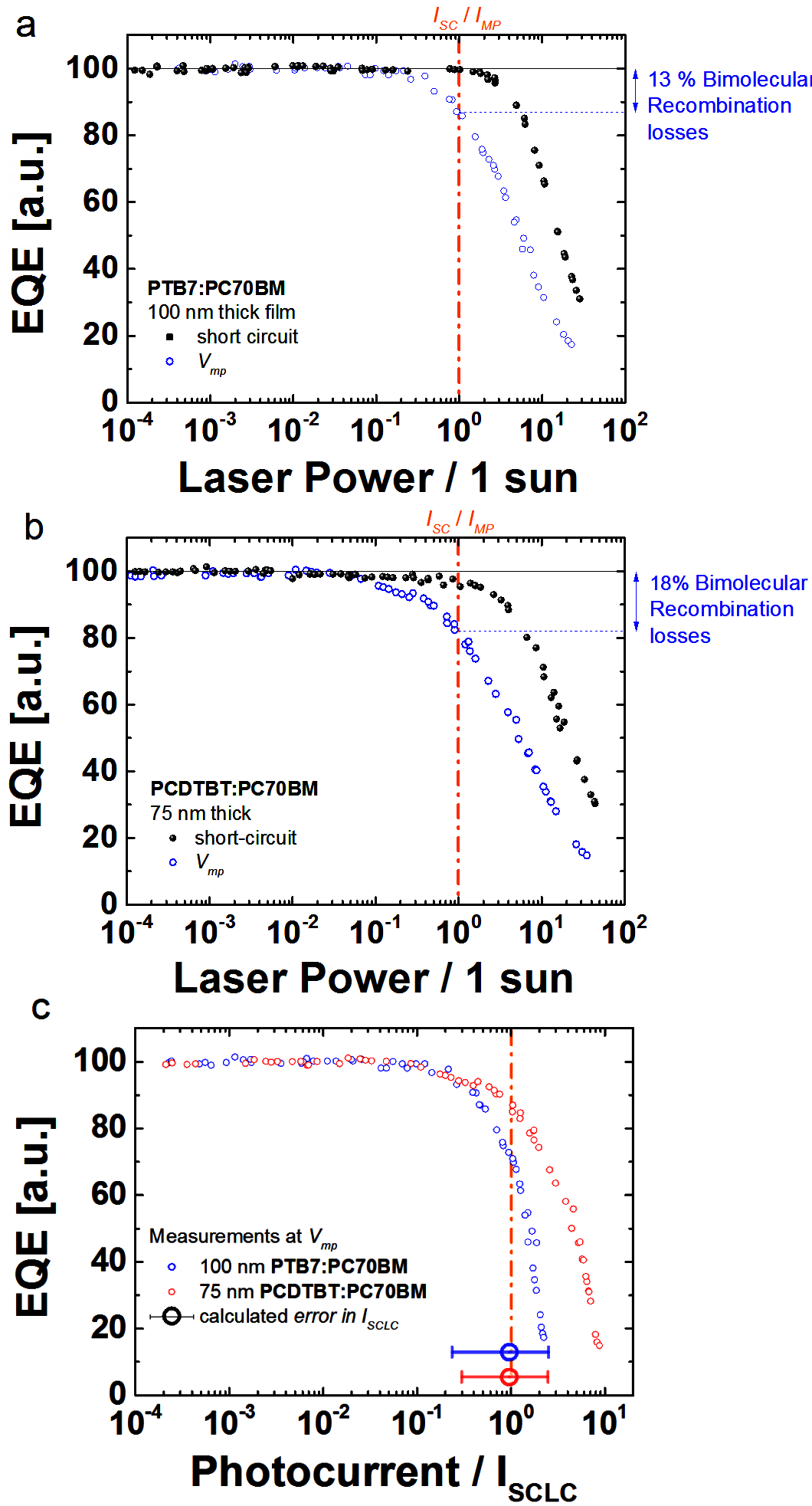

**Supplementary Figure 9.** External Quantum Efficiencies (EQEs) normalized to 100% shown as a function of the laser power (normalized by the 1 sun equivalent power) at the operational maximum power point conditions (*V*_mp_) in the highest efficiency (a) PTB7:PC70BM (100 nm) and (b) PCDTBT:PC70BM (75 nm) devices. The results are compared to the measurements at short-circuit conditions taken from **Figure 3** of the main text. While bimolecular recombination is not a significant problem in the optimized solar cells, at short-circuit, these losses are larger at *V*_mp_. (c) The photocurrent dependent EQEs show that the bimolecular recombination onset is also at *V*_mp_ governed by the *I*_SCLC_. Note, that the sharp EQE decrease in Figure (c) after the deviation in PTB7:PC70BM blend with 100 nm junction thickness is caused by the series resistance of indium tin oxide (see **Supplementary Note 2** and **Supplementary Figure 11**)


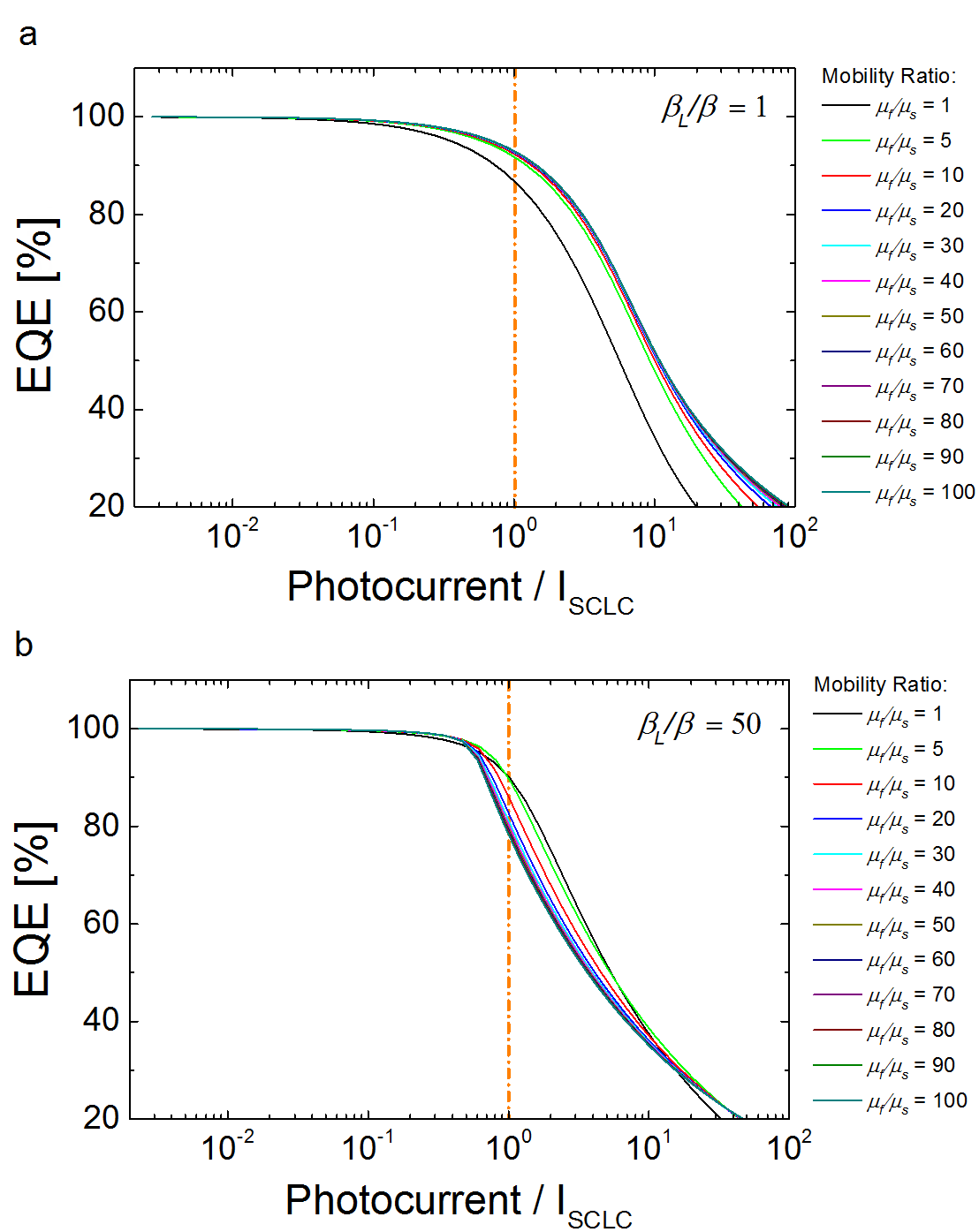


**Supplementary Figure 10.** Simulated external quantum efficiencies (EQEs) as a function of the photocurrent for a Langevin recombination rate (a) and suppressed Non-Langevin recombination *β*_L_*/β* = 50 (b) for various mobility ratios. In both graphs the faster carrier mobility is increased (up to 100 times), while the slower carrier mobility is kept constant. It can be seen that the onset of the recombination is determined by the *μ*_s_ (*β*_L_/*β*)^1/2^ product regardless of the faster carrier mobility (only small benefits of more balanced mobilities can be observed in graph (b)). Therefore, the simulations predict that it is not essential to balance the charge carrier mobilities for efficient charge extraction, which is in sharp contrast to what is commonly published in the literature [5, 6, 7]. The simulations also explain the exact onset position of the bimolecular recombination losses, which appears slightly earlier compared to the *I*_SCLC_ somewhat depending on the bimolecular coefficient ratio (*β*_L_/*β*).

**Supplementary Note 2.** Since the total circuit resistance (*R*_circuit_) can limit the photocurrent, it is critically important to know its impact on the iPC results. The impact of *R*_circuit_ was observed in the 100 nm PTB7:PC70BM blend (**Figure 4** of the main text), where the EQE deviates at a lower photocurrent compared to the *I*_SCLC_. This deviation, however, is not related to the charge transport parameters because the series resistance of indium tin oxide (ITO ~ 20 Ω) limits the photocurrent. Therefore, the maximum achievable photocurrent of this device with a built-in voltage (*U*_BI_) of ~ 0.7 V is around 35 mA, while the calculated *I*_SCLC_ for this device is much higher (~ 78 mA). Note, that the impact the finite *R*_circuit_ can be also observed in the other devices at higher photocurrents (> *I*_SCLC_) by the sharp decrease of the EQE.


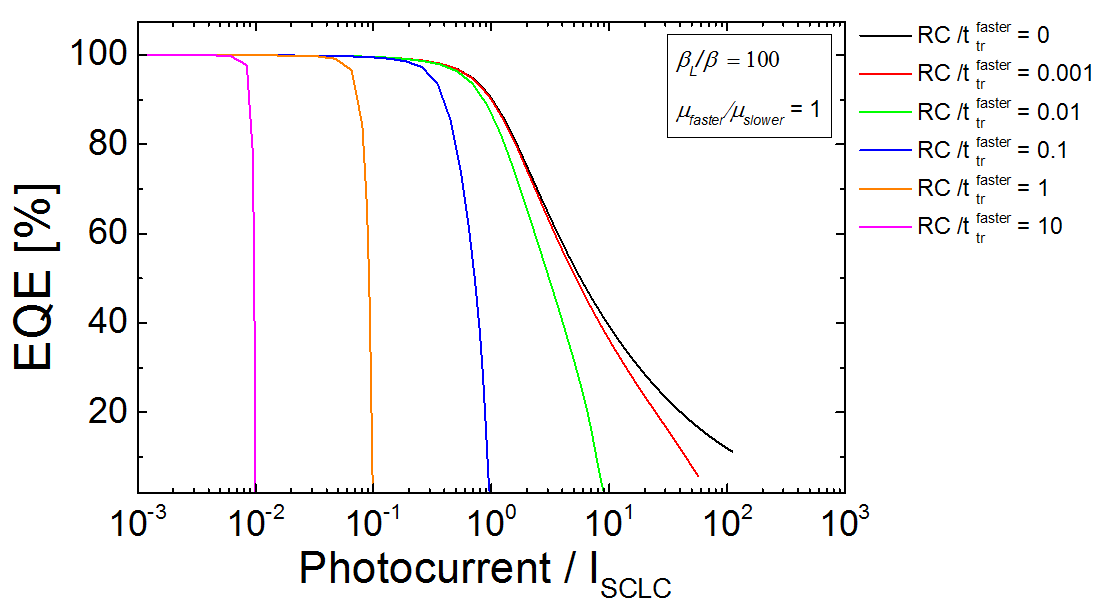


**Supplementary Figure 11.** The simulated impact of the circuit resistance on the IPC results. The graph shows a very steep decrease in the EQE when the photocurrent reaches approximately (~ 0.3 0.5 of) the saturation photocurrent (determined by *U*_BI_ / *R*_circuit_) if only the circuit resistance limits the extraction (pink and orange curve). By comparing the expected impact of the circuit resistance with the experimentally measured EQEs (**Figure 4** main text) we can exclude that the recombination onsets are caused by the circuit resistance in the studied devices (except the 100 nm PTB7:PC70BM blend ). We note, that the resistance-capacitance (*RC*) time constant in our devices is comparable to the faster carrier transit time and falls within the range shown in the legend.

**Supplementary Note 3.** To identify whether IPC results obtained upon monochromatic (continuous wave) laser illumination are still relevant for the device performance under standard AM 1.5G solar spectrum irradiation wavelength dependent iPC measurements are shown below.


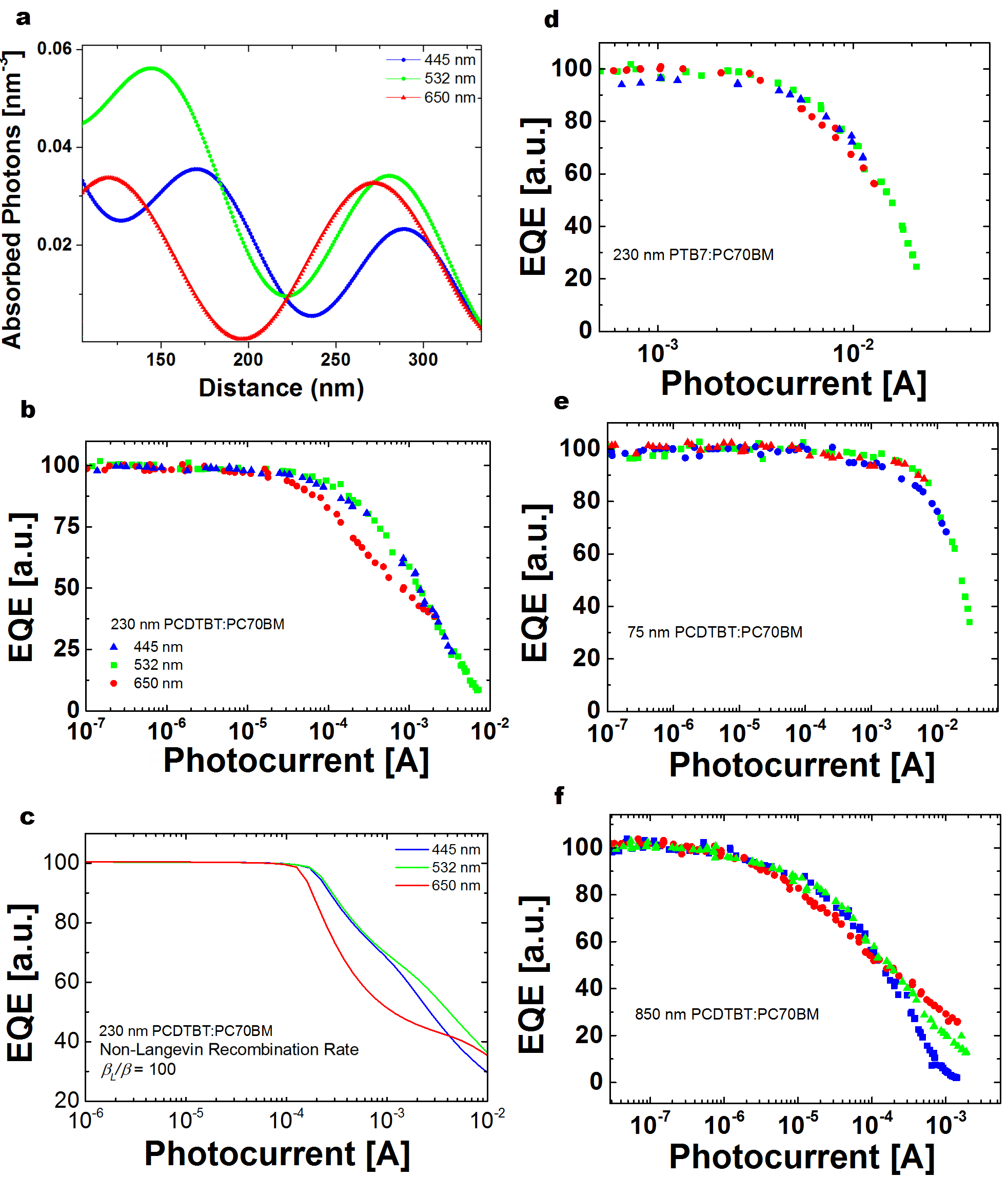


**Supplementary Figure 12.** The impact of the interference governed absorption profile (a) on the IPC results exemplified in (b) for the 230 nm thick PCDTBT:PC70BM blend. The absorption was simulated according to the transfer matrix method [8]. (c) The simulations were performed with realistic assumptions (that is: a Non-Langevin recombination rate, 100 times imbalanced mobilities with faster electrons). The graphs (a,b,c) demonstrate that even very different absorption profiles have a small impact on the onset of the bimolecular recombination losses. This demonstrates that iPC can be performed using one wavelength and the obtained results are indeed relevant to the operational AM 1.5G standard illumination. Nevertheless, we do not exclude that strongly asymmetric carrier distribution profiles can considerably affect the onset of the bimolecular recombination (as reported in [9]). If desired, iPC could be performed with a strong enough white LED – as opposed to a monochromatic laser - to be even closer to operational illumination conditions.

Irrelevant for this study, the differences in the sub-linear iPC regime, as measured and predicted by the simulations, are dependent on various experimental conditions. Notably, a wavelength dependence of the sub-linear iPC shape was observed in the experiment (in Fig. (b)), showing higher losses when the 650 nm laser is used. (d,e,f) Only small differences in the wavelength dependent bimolecular recombination losses were observed in other studied polymer:PC70BM blends for the 3 used lasers. Even in the thickest PCDTBT:PC70BM blend no differences were observed.


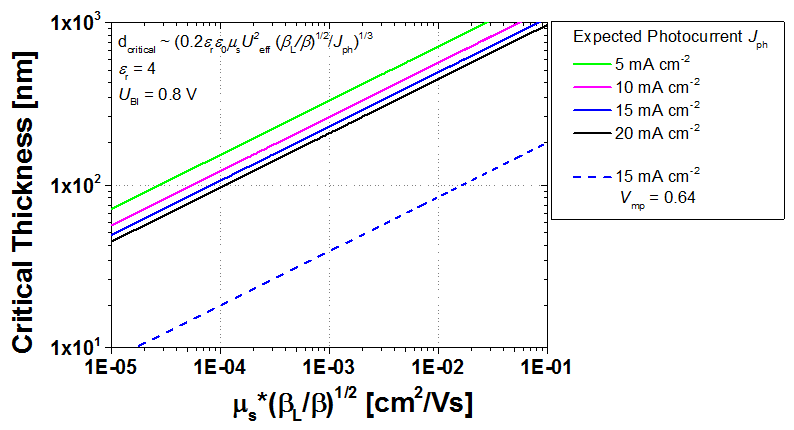


## **Supplementary Figure 13.** The active layer thickness required to avoid the bimolecular recombination losses plotted as a function of the slower carrier mobility times the square root of the bimolecular recombination coefficient ratio *μ*_s_ (*β*_L_/*β*)^1/2^ for different expected photocurrents. The presented experimental results and numerical simulations in this work demonstrate that the *I*_SCLC_ ~ *μ*_s_ (*β*_L_/*β*)^1/2^ must be higher than the expected (or maximum producible) photocurrent to avoid bimolecular recombination losses. Note, the appearance of the first bimolecular losses appears slightly earlier than the *I*_SCLC_ (see **Figure 4** of the main text and **Supplementary Figure 10**), therefore the *I*_SCLC_ has been multiplied by a constant of 0.2. (This factor was obtained as a rough estimation to describe the first recombination losses in the simulated Langevin and non-Langevin systems.) The results predict insufficient PC70BM electron mobilities (~ 3x10^-3^ cm^2^ V^-1^s^-1^, see below) to realize high fill factors in commercially relevant active layer thicknesses [10].


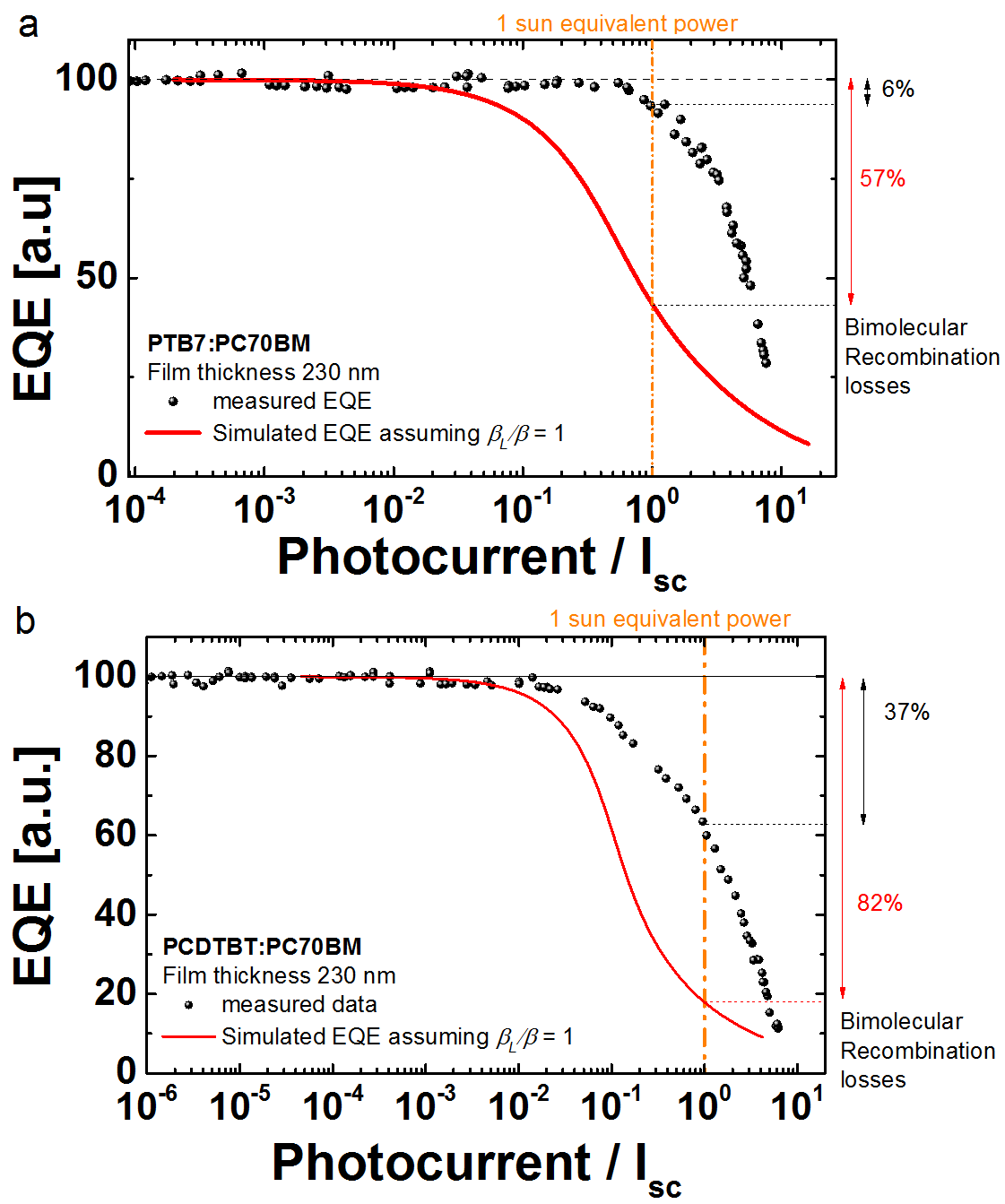


**Supplementary Figure 14.** A comparison between the experimentally measured photocurrent dependent EQEs with numerically simulated EQEs by using a Langevin recombination rate (*β*_L_/*β* = 1) for the PTB7:PC70BM (a) and PCDTBT:PC70BM blends (b) with active layer thicknesses of 230 nm. Note, the photocurrent was normalized to the short-circuit current (*I*_SC_) and the EQEs to 100%. Remarkable is the significantly prolonged recombination onset in both blends and the reduced recombination losses at the short-circuit conditions due to the non-Langevin behaviour.

**Supplementary Methods**

Simulations were performed using one dimensional continuity equations for electron and hole number densities. All quantities are scaled such that they are dimensionless, where dimensionless quantities are denoted with a prime.

Distances are scaled to the film thickness: $x^{'}\equiv x/d$. Times are scaled to the transit time calculated for the fastest mobility: $t^{'}\equiv t/t_{tr}$. The voltage scale is the internal voltage: $U^{'}\equiv U/U_{internal}$, where $U_{internal}$ is the sum of the built in voltage and the applied voltage. This system of units requires that the normalized faster carrier mobility is $\mu_{faster}^{'}=1$.

Charge is normalized to the charge on the electrodes $CU$, while number density is normalized to $CU$ per volume: $n^{'}\equiv enSd/CU$, where $S$ is the surface area of the device. Current is normalized to $CU$ per transit time: $i^{'}\equiv it_{tr}/CU$. The circuit resistance is $R^{'}\equiv RC/t_{tr}$.

The Einstein relation for diffusion gives a dimensionless temperature $T=kT/eU_{internal}$. The recombination coefficient is normalized to the Langevin rate: $\beta^{'}\equiv\beta/\beta_{L}$.

In the above system of units, the equations become:

|  | $j_{p}^{'}=\mu_{p}^{'}E^{'}p^{'}-\mu_{p}'T'\frac{\partial p^{'}}{\partial x^{'}}$ |  |
| --- | --- | --- |
|  | $j_{n}^{'}=\mu_{n}^{'}E^{'}n^{'}+\mu_{n}'T'\frac{\partial n^{'}}{\partial x^{'}}$ |  |
|  | $\frac{\partial p^{'}}{\partial t^{'}}+\frac{\partial j_{p}^{'}}{\partial x^{'}}=-\beta\left( \mu_{p}^{'}+\mu_{n}^{'} \right)n^{'}p^{'}$ |  |
|  | $\frac{\partial n^{'}}{\partial t^{'}}-\frac{\partial j_{n}^{'}}{\partial x^{'}}=-\beta\left( \mu_{p}^{'}+\mu_{n}^{'} \right)n^{'}p^{'}$ |  |
|  | $\frac{\partial^{2}U^{'}}{\partial\left( x^{'} \right)^{2}}=n^{'}-p^{'}$ |  |
|  | $E^{'}=-\frac{\partial U^{'}}{\partial x^{'}}.$ |  |

The boundary conditions for Poisson’s equation are:

|  | $U^{'}\left( t,0 \right)=V'$ |  |
| --- | --- | --- |
|  | $U^{'}\left( t,1 \right)=0,$ |  |

where $V^{'}$ is the voltage across the semiconductor:

|  | $\frac{dV^{'}}{dt^{'}}=\frac{1-V'}{R^{'}}-j_{c}'$ |  |
| --- | --- | --- |
|  | $j_{c}^{'}=\int_{0}^{1} j_{p}^{'}\left( x \right)+j_{n}^{'}\left( x \right) dx.$ |  |

We use a finite volume method, so the boundary conditions for the transport equations are expressed in terms of the fluxes $j_{p}^{'}$ and $j_{n}'$ at each electrode (a total of four fluxes, for two types of carrier each at two boundaries). Since the experiment is conducted under reverse bias, we assume no injection is possible. This immediately sets two such edge fluxes to zero. The other two represent charge *extraction* and are described by the local drift current $j_{p}^{'}=\mu_{p}^{'}E'p'$ (and similarly for electrons).

The initial condition for the number density in the Beer-Lambert case is:

|  | $n^{'}\left( 0,x^{'} \right)=p^{'}\left( 0,x^{'} \right)=L^{'}\alpha^{'}e^{-\alpha^{'}x^{'}},$ |  |
| --- | --- | --- |

with $Q_{ph}^{'}=L^{'}\left( a-e^{-\alpha^{'}} \right)$ and $\alpha^{'}\equiv\alpha d;$ or alternatively, by the condition of uniform generation

|  | $n^{'}\left( 0,x^{'} \right)=p^{'}\left( 0,x^{'} \right)=Q_{ph}.$ |  |
| --- | --- | --- |

The initial condition for voltage is $V^{'}=1.$

**Supplementary References**

[1] Philippa, B. et al. The impact of hot charge carrier mobility on photocurrent losses in polymer-based solar cells. *Sci. Rep.* **4**, 1–8 (2014).

[2] Stolterfoht, M. et al. Advantage of suppressed non-langevin recombination in low mobility organic solar cells. *Appl. Phys. Lett.* **105**, 013302 (2014).

[3] Juška, G. et al. Charge transport in pi-conjugated polymers from extraction current transients. *Phys. Rev. B Condens Matter.* **62**, R16235–R16238 (2000).

[4] Philippa, B. et al. Molecular weight dependent bimolecular recombination in organic solar cells. *J. Chem. Phys.* **141**, 054903 (2014).

[5] Mihailetchi, V. D., Wildeman, J., and Blom, P. W. M. Space-charge limited photocurrent. *Phys. Rev. Lett.* **94**, 126602 (2005).

[6] Kotlarski, J. D., Moet, D. J. D., and Blom, P. W. M. Role of balanced charge carrier transport in low band gap polymer:fullerene bulk heterojunction solar cells. *J. Polym. Sci. Pol. Phys.* **49**, 708–711 (2011).

[7] Mandoc, M. M., Koster, L. J. A., and Blom, P. W. M. Optimum charge carrier mobility in organic solar cells. *Appl. Phys. Lett.* **90**, 133504 (2007).

[8] Peumans, P., Yakimov, A., and Forrest, S. R. Small molecular weight organic thin-film photodetectors and solar cells. *J. Appl. Phys.* **93**, 3693–3723 (2003).

[9] Tumbleston, J. R., Liu, Y., Samulski, E. T., and Lopez, R. Interplay between bimolecular recombination and carrier transport distances in bulk heterojunction organic solar cells. *Adv. Energy Mater.* **2**, 477–486 (2012).

[10] Krebs, F. C. Fabrication and processing of polymer solar cells: A review of printing and coating techniques. *Sol. Energ. Mat. Sol. C.*  **93**, 394 – 412 (2009).
